# Ciliary parathyroid hormone signaling activates transforming growth factor-β to maintain intervertebral disc homeostasis during aging

**DOI:** 10.1038/s41413-018-0022-y

**Published:** 2018-07-18

**Authors:** Liwei Zheng, Yong Cao, Shuangfei Ni, Huabin Qi, Zemin Ling, Xin Xu, Xuenong Zou, Tianding Wu, Ruoxian Deng, Bo Hu, Bo Gao, Hao Chen, Yusheng Li, Jianxi Zhu, Francis Tintani, Shadpour Demehri, Amit Jain, Khaled M. Kebaish, Shenghui Liao, Cheryle A. Séguin, Janet L. Crane, Mei Wan, Hongbin Lu, Paul D. Sponseller, Lee H. Riley, Xuedong Zhou, Jianzhong Hu, Xu Cao

**Affiliations:** 1 Department of Orthopedic Surgery, Johns Hopkins University School of Medicine, Ross Building, Room 229, 720 Rutland Ave, Baltimore, MD USA; 2 State Key Laboratory of Oral Diseases, West China Hospital of Stomatology, Sichuan University, Chengdu, China; 3 Department of Spine Surgery, Xiangya Hospital, Central South University, Changsha, China; 4 Center of Bone Metabolism and Repair (CBMR), Trauma Center, Institute of Surgery Research, Daping Hospital, Third Military Medical University, Chongqing, China; 5 Guangdong Provincial Key Laboratory of Orthopedics and Traumatology, Department of Spinal Surgery, The First Affiliated Hospital of Sun Yat-sen University, Guangzhou, China; 6 Department of Pediatrics, Johns Hopkins University School of Medicine, Ross Building, Room 229, 720 Rutland Ave, Baltimore, MD USA; 7 The Russell H. Morgan Department of Radiology and Radiological Science, Johns Hopkins University School of Medicine, Ross Building, Room 229, 720 Rutland Ave, Baltimore, MD USA; 8 Faculty of Computer Science, College of Information Science and Engineering, Xiangya Hospital, Central South University, Changsha, China; 9 Department of Physiology and Pharmacology, Schulich School of Medicine and Dentistry, University of Western Ontario, London, ON Canada; 10 Department of Sports Medicine, Xiangya Hospital, Central South University, Changsha, China

## Abstract

Degenerative disc disease (DDD) is associated with intervertebral disc degeneration of spinal instability. Here, we report that the cilia of nucleus pulposus (NP) cells mediate mechanotransduction to maintain anabolic activity in the discs. We found that mechanical stress promotes transport of parathyroid hormone 1 receptor (PTH1R) to the cilia and enhances parathyroid hormone (PTH) signaling in NP cells. PTH induces transcription of integrin α_v_β_6_ to activate the transforming growth factor (TGF)-β-connective tissue growth factor (CCN2)-matrix proteins signaling cascade. Intermittent injection of PTH (iPTH) effectively attenuates disc degeneration of aged mice by direct signaling through NP cells, specifically improving intervertebral disc height and volume by increasing levels of TGF-β activity, CCN2, and aggrecan. PTH1R is expressed in both mouse and human NP cells. Importantly, knockout PTH1R or cilia in the NP cells results in significant disc degeneration and blunts the effect of PTH on attenuation of aged discs. Thus, mechanical stress-induced transport of PTH1R to the cilia enhances PTH signaling, which helps maintain intervertebral disc homeostasis, particularly during aging, indicating therapeutic potential of iPTH for DDD.

## Degenerative disc disease: hormone signaling keeps intervertebral core in balance

Sensory structures found in the jelly-like space between spinal discs release a hormone that helps preserve back health in aging mice. Xu Cao from Johns Hopkins University in Baltimore, Maryland, USA, and colleagues observed that levels of a critical growth factor declined in the space between adjacent vertebrae as mice aged, and that injecting a naturally occurring hormone that activates this growth factor could attenuate disc degeneration in older animals. The researchers showed, in response to mechanical stresses, receptor proteins that respond to this hormone relocate themselves to particular sensory organelles known as cilia that found within cells of the intervertebral core. That results in elevated hormone signaling—and drugs designed to have the same effect could help treat degenerative disc disease, one of the most common causes of chronic back pain.

## Introduction

Degenerative disc disease (DDD) is one of the most common musculoskeletal disorders as the leading cause of disability.^1,2^ Disc degeneration has been detected as early as adolescence, and 60% of people aged 70 years have severe disc degeneration.^3–5^ Despite the prevalence of DDD, we still have no effective disease-modifying therapy, largely due to our limited knowledge about mechanisms of intervertebral disc homeostasis and degeneration. The cost of the disease is over $100 billion annually in the US alone, more than stroke, respiratory infection, diabetes, coronary artery disease, and rheumatoid disease combined.^3–5^ The current treatments are primarily on symptomatic relief from pain through injections, physical therapy, and activity modification^6^ or surgical intervention, such as disc decompression, spinal fusion, and disc replacement.^1,5^ These interventions do not halt progression of degeneration nor restore the physiologic disc function.

Intervertebral disc consists of gel-like nucleus pulposus (NP) in the central compartment surrounded by an outer network of collagen as the annulus fibrosus (AF). Its function as pads between vertebral bodies is to absorb axial compressive forces transmitted along the spine while permitting flexibility in bending and twisting. The NP cells of notochordal origin synthesize extracellular matrix (ECM) (primarily proteoglycans and fine collagen type-II fibrils) in response to hydrostatic pressure, whereas cells in AF synthesize mostly collagen type I in response to the deformation in the maintenance of disc homeostasis.^7,8^ During aging, the cell density, proteoglycan, and water content in the disc decreases, especially in the nucleus.^9^ Disc degeneration is a cell-mediated progressive structural failure with accelerated signs of aging, and early degenerative changes are primarily accelerated sign of aging in a structurally intact disc.^9^

Intervertebral disc cells embedded in the different areas are exposed to wide ranges of mechanical loads. NP cells are located at the center of discs with higher water content and the types of in vivo loading are hydrostatic pressure, as well as compressive load and shear stress due to the swelling properties of NP tissue,^10^ whereas cells in AF are exposed predominantly to tensile strain. Disc cells are able to sense mechanical stress and transform it into biological signals to maintain homeostasis of intervertebral discs.^11–13^ This mechanotransduction enables cells to translate different physical forces into biochemical activities in regulation of tissue function, whereas aberrant mechanotransduction often lead to pathological changes or degenerative states.^11^ The mechanisms of mechanotransduction has been extensively studied in osteoblasts and chondrocytes. The transmembrane calcium ion channels, receptor tyrosine kinases (RTK), and integrins appear to mediate mechanotransduction in these cells.^14^ However, the knowledge of mechanotransduction in disc cells is rather limited relative to bone and chondrocytes. Certain integrins have been implicated in the compressive loading-induced down-regulation of aggrecan gene expression in human NP cell cultures with an Arg-Gly-Asp (RGD)-inhibitory peptide prior to compression.^15^ Active transforming growth factor (TGF)-β is known to act upstream of connective tissue growth factor (CTGF/CCN2) and aggrecan, both of which are involved in DDD development.^16,17^ We have recently found that mechanical loads induce integrin α_v_β_6_-mediated activation of TGFβ, which in return induces transcription of connective tissue growth factor (CTGF/CCN2) to increase matrix proteoglycan production.^18^ Knockout of TGFβ type II receptor (TβRII) or integrin αv in the NP cells results in disc degeneration. Thus, integrin-mediated activation of TGFβ in response to mechanical loads maintains disc function and homeostasis.

The primary cilium is the immotile microtubule-based extensions as a single cellular protrusion presenting on nearly every mammalian cell. Primary cilium functions as a mechanosensor and dynamic signaling organelle that projects from the cell surface to sense microenvironment changes including diverse signal molecules and chemicals.^19^ The specialized subcellular compartment of a cilium concentrates membrane-bound receptors to effectively detect mechanical stimuli, as well as sensory inputs and ligands from the extracellular environment for the cellular responses.^20,21^ Recent studies revealed that the vertebrate cell requires a single non-motile primary cilium to respond to Hedgehog (HH) family ligands.^22,23^ Interestingly, many G protein-coupled receptors (GPCRs) are found to signal effectively in the cilia.^24,25^ Cells with cilia defects cannot sense the changes in mechanical environment and are associated with numerous human diseases including spinal deformity.^26,27^ NP cells stimulate anabolic synthesis of ECM proteins in the disc in response to mechanical stress. It is believed that cilia have a critical role in intervertebral disc function although there is no report regarding cilia on NP cells.

Parathyroid hormone (PTH) secreted by the parathyroid gland binds to the type 1 PTH receptor (PTH1R), a GPCR and stimulates adenylate cyclase for formation of cyclic adenosine monophosphate (cAMP).^28^ Increased cAMP levels activate protein kinase A (PKA) to induce phosphorylation of cAMP response element binding (CREB) protein, a transcription factor responsible for PTH downstream gene expression.^29^ PTH stimulates bone remodeling and orchestrates signaling of multiple growth factors and cytokines including TGF-β, Wnts, bone morphogenetic protein (BMP), and Insulin-like growth factor (IGF-1).^30–34^ PTH also spatially relocates small blood vessels closer to sites of new bone formation.^35,36^ The closer proximity of blood vessels allows more efficient delivery of nutrients for new bone formation. PTH emerges during the evolution of amphibians as they adapted to terrestrial life, indicating a potential role of PTH in spinal activity, particularly in intervertebral disc function. In this study, we sought to investigate how mechanotransduction in NP cells maintains the anabolic activity in the intervertebral disc. Our findings reveal that ciliary PTH signaling enables mechanotransduction for disc anabolic activity.

## Results

### TGF-β activity decreased during disc degeneration of aging

**Fig. 1:**
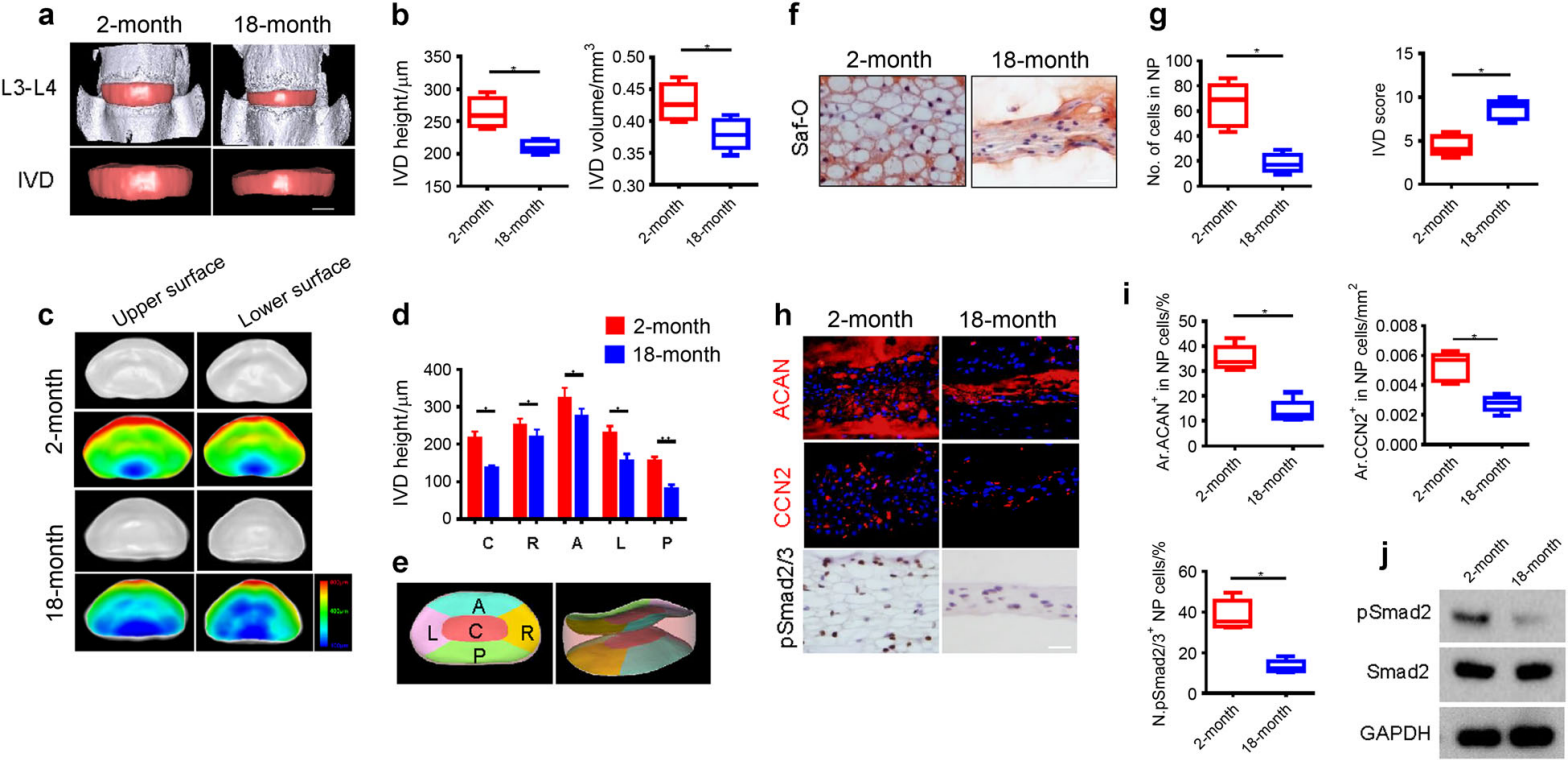
IVD volume and TGF-β activity decrease during aging. **a** 3D propagation phase contrast micro-tomography (PPCT) images of IVDs of 2-month and 18-month-old mice. Scale bars, 500 μm. **b** Quantitative analysis of IVD height and volume. **c** 3D upper and lower surface PPCT images showing thickness distribution among five areas of IVDs in 2-month and 18-month old mice and (**d**) quantitative analysis of (**c**). Scale bar, color code indicating degree of thickness from blue (100 μm) to red (800 μm). **e** A 3D PPCT image of IVD of lumbar 3rd and 4th was randomly selected to draw this schematic diagram to define the five regions with labeling on the IVD including C: central region, R: right region, A: anterior region, L: left region, P: posterior region based on the anatomical characters. **f**, **g** Safranin-O staining images of IVD tissue sections showing nucleus pulposus (NP) area (**f**) and quantitative analysis of the cell numbers in NP area and IVD histological scores of 2-month and 18-month old mice (**g**). Scale bars, 100 μm. **h** Immunostaining images of IVD sections showing expression of aggrecan (ACAN), CCN2, and pSmad2/3 positive cells in the NP area. Scale bars, 200 μm. **i** Quantitative analysis of ACAN-positive and CCN2-positive areas as percentage of total IVD area and pSmad2/3-positive cells in NP area (Ar). Scale bars, 50 μm. **j** Western blot analysis showing pSmad2 levels in NP tissues of 2-month and 18-month old mice. All data shown as mean ± s.d. *P < 0.05, **P < 0.01, *n* = 8 per group. Statistical significance was determined by Student's *t*-test

We systematically examined the changes of intervertebral disc during aging. Three dimensional (3D) changes of the intervertebral disc were visualized using propagation phase contrast micro-tomography (PPCT) based on synchrotron radiation showing 3D images of the intervertebral disc with adjoining vertebra (top) and intact intervertebral disc (bottom) in 2-month and 18-month-old mice (Supplementary Fig. 1 and Fig. 1a). The height and volume of the intervertebral discs were significantly decreased in 18-month-old mice relative to 2-month-old mice (Fig. 1b). Similarly, thickness decreased with aging in five different intervertebral disc areas (Fig. 1c–e and Supplementary Fig. 1), especially the posterior area. The number of NP cells also significantly decreased as visualized by Safranin-O staining of intervertebral disc sections in 18-month-old mice with an increase in intervertebral disc score indicating degeneration (Fig. 1f, g). Immunohistochemical staining demonstrated that the levels of aggrecan (ACAN), CTGF/CCN2^+^, and pSmad2/3^+^ cells in the intervertebral disc were significantly decreased in 18-month-old mice relative to 2-month-old mice (Fig. 1h, i). Western blot analysis validated the decrease of pSmad2/3 in the 18-month-old mice (Fig. 1j). Our results suggested that the decrease of active TGF-β is associated with its downstream anabolic CCN2-ECM cascade during age-related intervertebral disc degeneration.

### PTH directly induces cAMP production and phosphorylation of CREB (pCREB) in NP cells

**Fig. 2:**
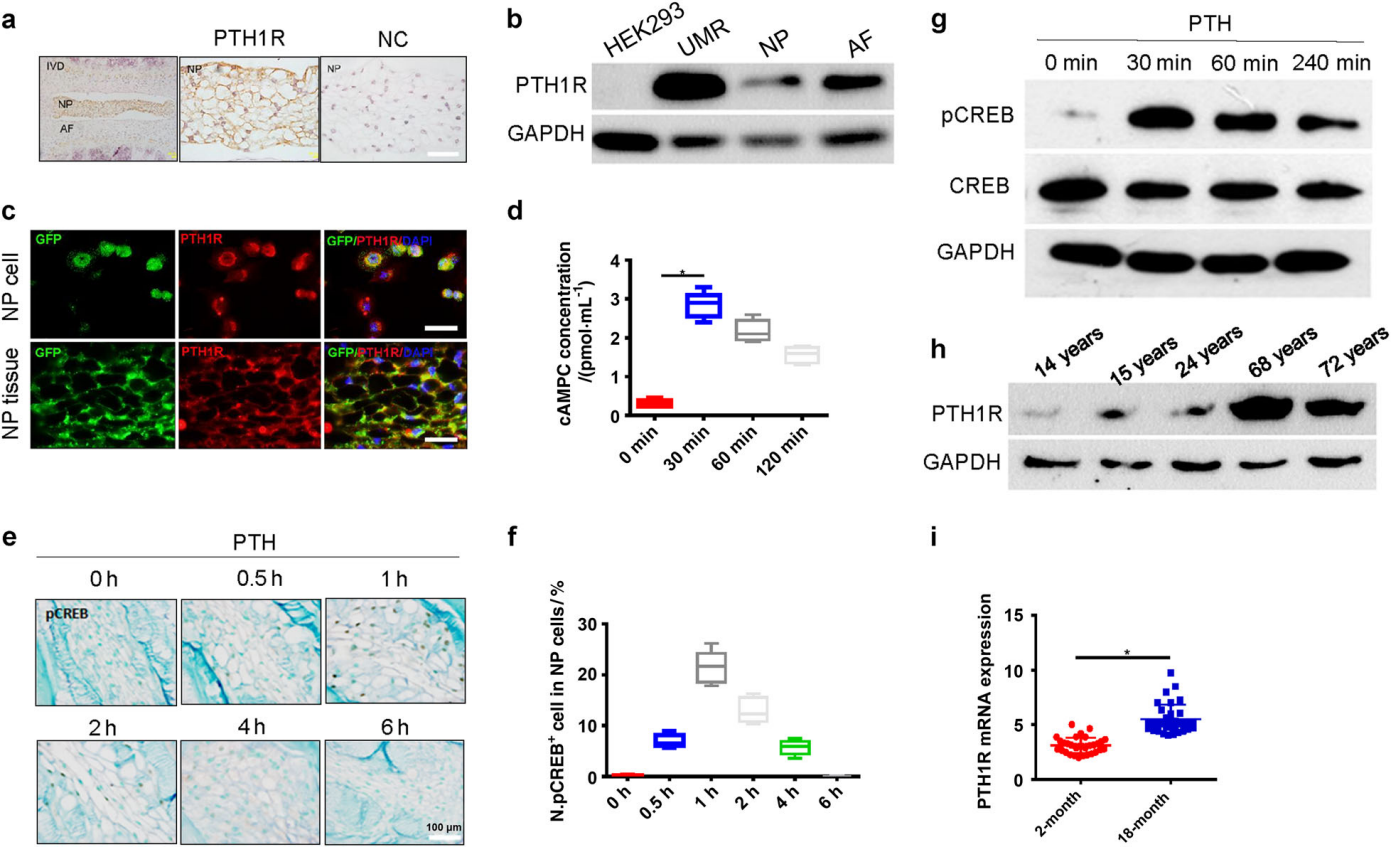
PTH directly induces cAMP production and phosphorylation of CREB in NP cells. **a** Immunostaining images of mouse IVD sections showing PTH1R (brown) in NP cells with IgG antibody as negative control (NC). Scale bar, 100 μm. **b** Western blot analysis showing PTH1R expression in NP and AF cells with HEK293 cells as negative control (NC) and UMR-106 osteoblast-like cells as positive control (PC). **c** Lineage mapping of PTH1R expression in NP cells of notochordal origin (top yellow) using *Noto*^*Cre*^; ROSA26-GFP mice. Scale bar, 20 μm (top) and 50 μm (bottom). NP cells stained positively for notochord origin (green) and presence of PTH1R receptor (red). Cells of notochordal origin (green) and PTH1R positive cells (red) stained abundantly with in NP area of disc tissue co-localization (yellow). **d** ELISA analysis of cellular cAMP levels in NP cells with PTH treatment. **e** Immunostaining of IVD sections showing pCREB-positive cells in NP area at different time points with PTH treatment. Scale bars, 100 μm. **f** Percentage of pCREB-positive cells versus total NP cells with PTH treatment. **g** Western blot analysis of pCREB levels in NP cells with PTH treatment. **h** Western blot analysis of PTH1R expression in human NP specimens at different ages. **i** qRT-PCR of PTH1R mRNA levels in NP tissues from young and older patients shown as fold changes. All data shown as mean ± s.d. *P < 0.05, **P < 0.01. *n* = 8 per group. Statistical significance was determined by Student's *t*-test

We examined whether PTH activates its downstream signaling directly in NP cells as PTH gland evolved in amphibians suggests its function for adaptation of vertebrates on land. Immunostaining of L_3_–L_4_ disc sections showed that PTH1R was expressed in NP cells (brown area) (Fig. 2a). Western blot analysis further demonstrated the expression of PTH1R in NP and AF (Fig. 2b). To validate that PTH1R expression in NP cells is of notochordal origin, we crossed ROSA26-GFP^flox/flox^ mice with Noto^Cre^ mice to generate mice with green fluorescent protein (GFP) fluorescence in the notochord cell lineage (Noto^Cre^::ROSA26-GFP). GFP^+^ intervertebral disc cells isolated from the Noto^Cre^::ROSA26-GFP mice co-localized with PTH1R (Fig. 2c, upper panel) and immunofluorescence staining of the intervertebral disc sections also demonstrated that PTH1R was co-localized with GFP^+^ cells in the NP area (Fig. 2c, lower panel),confirming that PTH1R is expressed in the notochord-derived NP cells.

To elucidate whether PTH stimulates downstream intracellular signaling, we measured the level of PTH-induced cyclic adenosine monophsphate (cAMP) production in NP cells. The cAMP level in NP cells peaked at 30 min with PTH (1–34) (100 nmol) treatment (Fig. 2d). The number of pCREB^+^ cells increased in NP area at 30 min and peaked at 1 h after treatment (Fig. 2e, f). Western blot analysis also demonstrated that PTH induced pCREB at 30 min in NP cells (Fig. 2g). Importantly, PTH1R protein levels in the human NP and AF cells were relatively low in young and adults but significantly increased during aging in Western blot analysis of patient lumbar intervertebral disc specimens (Fig. 2h). Similarly, the levels of PTH1R messenger ribonucleic acid (mRNA) expression in NP tissue were relatively low in young adults and increased with aging (Fig. 2i). Taken together, these results revealed the direct signaling of PTH in NP cells, suggesting its potential role in the maintenance of intervertebral discs during aging.

### Intermittent injection of PTH (iPTH) attenuates disc degeneration by inducing integrin α_v_β_6_ expression in activation of TGF-β

**Fig. 3:**
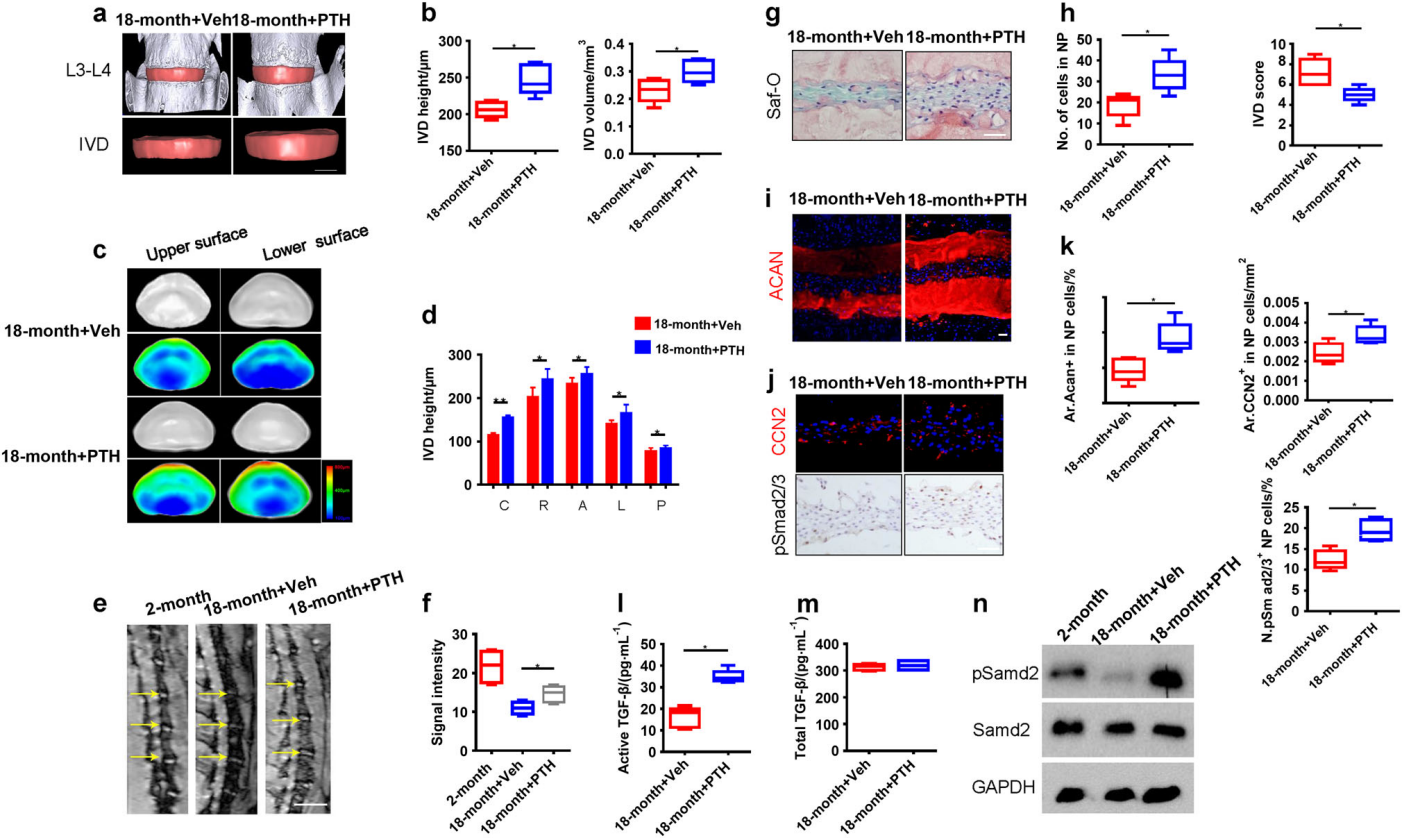
iPTH attenuates disc degeneration by activation of TGF-β. **a** 3D PPCT images of IVDs with iPTH (1–34) injection of 40 μg·kg^-1^ daily or vehicle of 18-month old mice, 5 days per week for 8 weeks. Scale bars, 500 μm. **b** Quantitative analysis of mouse IVD height and IVD volume. **c**,**d** 3D PPCT images and quantitative analysis showing thickness distribution of five regions of IVD treated with iPTH or vehicle. Color code indicating degree of thickness from blue (100 μm) to red (800 μm). **e**, **f** MRI scan of mouse lumbar spine showing signal intensity of the discs (yellow arrow) of 18-month old mice treated with iPTH or vehicle in comparison with MRI scan of 2-month old mice (**e**) and quantitative measurements of the disc signal intensity (**f**). Scale bars, 1 mm. **g**, **h** Safranin-O staining images of IVD sections showing NP area of 18-month old mice with iPTH or vehicle (**g**) and quantitative analysis of cell numbers in NP area and IVD histological scores (**h**). Scale bars, 100 μm. **i**, **j** Immunostaining images of IVD sections showing expression of ACAN, CCN2, and pSmad2/3 positive cells in NP area. Scale bars, 200 μm. **k** Quantitative analysis of the ACAN-positive, CCN2-positive areas and the number of pSmad2/3 positive cells as a percentage of total IVD area (Ar). **l**, **m** ELISA analysis of total and active TGF-β levels from NP tissue in 18-month old mice treated with iPTH or vehicle. **n** Western blot analysis showing pSmad2 levels on in NP tissues from 18-month-old mice injected with PTH or vehicle in comparison with that of NP tissues from 2-month old. All data are reported as the mean ± s.d. *P < 0.05, **P < 0.01. *n* = 8 per group. Statistical significance was determined by one-way ANOVA and Student's *t*-test

To examine the effect of iPTH on intervertebral disc degeneration, we injected aged mice with PTH, a C-terminal truncated synthetic analog of human PTH (1–34) daily for 8 weeks with different doses. iPTH doses of 40 and 80 µg·kg^-1^ significantly improved the intervertebral disc morphology and the dose of 40 µg·kg^-1^ was chosen for the rest of the study (Supplementary Fig. 2a, b). Specifically, intervertebral disc height and volume were increased with iPTH in the 18-month-old mice relative to vehicle mice (Fig. 3a, b), with increase of bone volume fraction (BV/TV) and trabecular connectivity (Tb. Con) (Supplementary Fig. 2c, d). Moreover, the significant increase was shown in intervertebral disc thickness in 3D images in five different areas of the disc with iPTH, especially the central area (Fig. 3c, d). The MRI signal intensity of lumbar intervertebral discs significantly increased with iPTH injection (Fig. 3e, f). The number of NP cells significantly increased with the decrease of intervertebral disc score (Fig. 3g, h) and the level of aggrecan and CCN2 increased with iPTH (Fig. 3i–k). Furthermore, the number of pSmad2/3^+^ NP cells increased with iPTH treatment (Fig. 3j, k), indicating that PTH induces activation of TGF-β in intervertebral disc. Indeed, the level of active TGF-β increased in intervertebral discs with iPTH treatment while total TGF-β level was not changed in ELISA (Fig. 3l, m). Western blot analysis confirmed that pSmad2 was increased with iPTH (Fig. 3n).

**Fig. 4:**
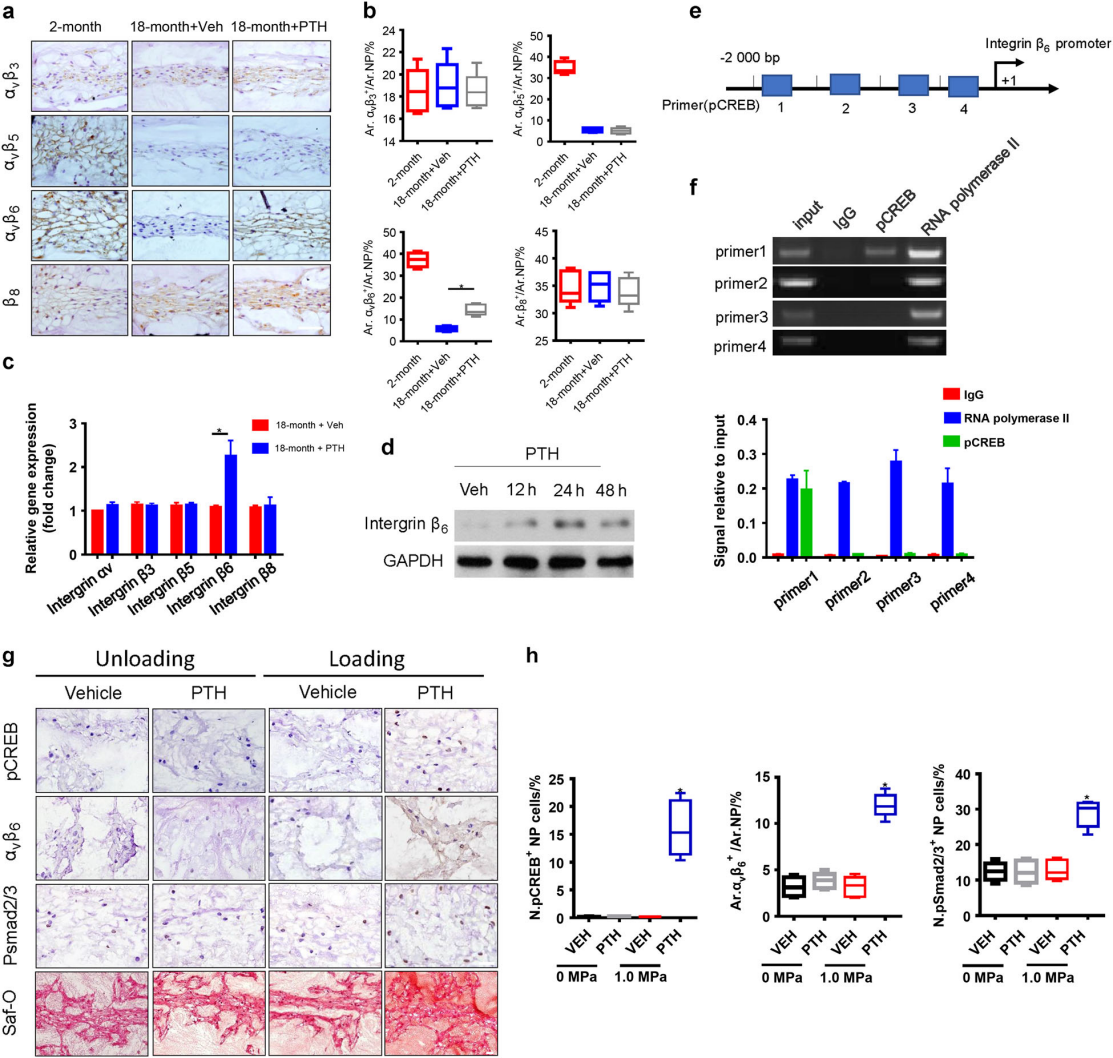
PTH induces integrin α_v_β_6_ expression to activate latent TGF-β. **a** Immunostaining images showing various types of integrin expressions in IVD tissue from 18-month-ld mice injected with PTH or vehicle and quantitative analysis (**b**). Scale bar, 50 μm. **c** qRT-PCR analysis of the mRNA levels of various integrin in NP tissue from 18-month-old mice injected with PTH or vehicle. Results reported as fold change. **d** Western blot analysis of integrin β_6_ expression in NP cells of 18-month-old mice at different time points post PTH injection (PTH1-34, 100 nmol·L^-1^). **e**, **f** Chromatin immunoprecipitation assay with four different potential pCREB binding sites (primers 1, 2, 3 and 4) in the β6 integrin promoter. **g** pCREB, Integrin α_V_β_6_, pSmad2/3, or Safranin-O staining of IVD sections from an IVD ex vivo compression model of 30-month-old rat with treatment of either vehicle or PTH (PTH1-34, 100 nmol·L^-1^). Scale bar, 20 μm. **h** Quantitative analysis of the percentage of pCREB, pSmad2/3 positive cells and the Integrin α_V_β_6_ positive areas as a percentage of total IVD area (Ar) of (**g**). All data are reported as the mean ± s.d. *P < 0.05. *n* = 8 per group. Statistical significance was determined by one-way ANOVA and Student's *t*-test

To determine the mechanism by which PTH induces the activation of TGF-β in intervertebral disc, we examined whether PTH increased the expression of α_v_β integrins, which mediate activation of latent TGF-β.^37–39^ Intervertebral disc sections prepared from iPTH-treated or vehicle-treated 18-month-old mice were immunostained with individual antibodies against α_v_β_3_, α_v_β_5_, α_v_β_6_, and β_8_ integrins, respectively. The results showed that the expressions of α_v_β_5_ and α_v_β_6_ integrins were significantly decreased in the 18-month-old mice relative to 2-month-old mice, while the expression levels of α_v_β_3_ and β_8_ remained relatively unchanged. Importantly, PTH only significantly increased the level of α_v_β_6_ in 18-month old mice (Fig. 4a, b). To validate the regulation of α_v_β_6_ integrin by iPTH, we performed quantitative real-time polymerase chain reaction (qRT-PCR) to quantify the effect of PTH treatment on individual integrin mRNA expression. Consistently, PTH specifically induced mRNA expression of β_6_ integrin in NP cells of the aged mice (Fig. 4c). PTH also significantly stimulated the protein level of β_6_ integrin in a time-dependent manner (Fig. 4d). Thus, PTH activates latent TGF-β by increasing α_v_β_6_ expression in NP tissue, offering a potential therapeutic target for disc degeneration.

To determine the mechanism of PTH-induced β_6_ integrin gene transcription, we performed chromatin immunoprecipitation (ChIP) assay with four different potential pCREB-binding sites (Supplementary Table 1: primers 1, 2, 3 and 4) in the β_6_ integrin promoter. Immunoprecipitation results revealed that pCREB specifically binds to the most distal CREB site in the β_6_-integrin promoter (Primer 1) (Fig. 4e, f). Finally, we validated the results in the 30-month-old rats as their disc cell densities were much lower during aging like NP cells in human discs. The L1–L5 lumbar IVDs were removed and cultured with loads of 0 or 1.0 MPa by vertically placing weights for 48 h with PTH or vehicle. The levels of CREB phosphorylation were significantly stimulated with PTH treatment and 1.0 MPa loads, whereas vehicle, loads, or PTH alone did not have the significant effect in both 3-month-old and 30-month-old aged rats (Fig. 4g, h and Supplementary Fig. 3). Similarly, PTH also stimulated levels of α_v_β_6_, pSmad2/3 and proteoglycan in Safranin O staining in the presence of loads. PTH stimulates its signaling in NP cells in mice as mechanical loads on discs always exist in vivo. This ex vivo IVD results revealed that mechanical loads are necessary for PTH signaling in NP cells. Taken together, these results demonstrated that PTH activates expression of β_6_ integrin by inducing direct binding of pCREB to the β_6_ integrin promoter in NP cells.

### Conditional knockout of PTH1R in NP cells accelerates disc degeneration

**Fig. 5:**
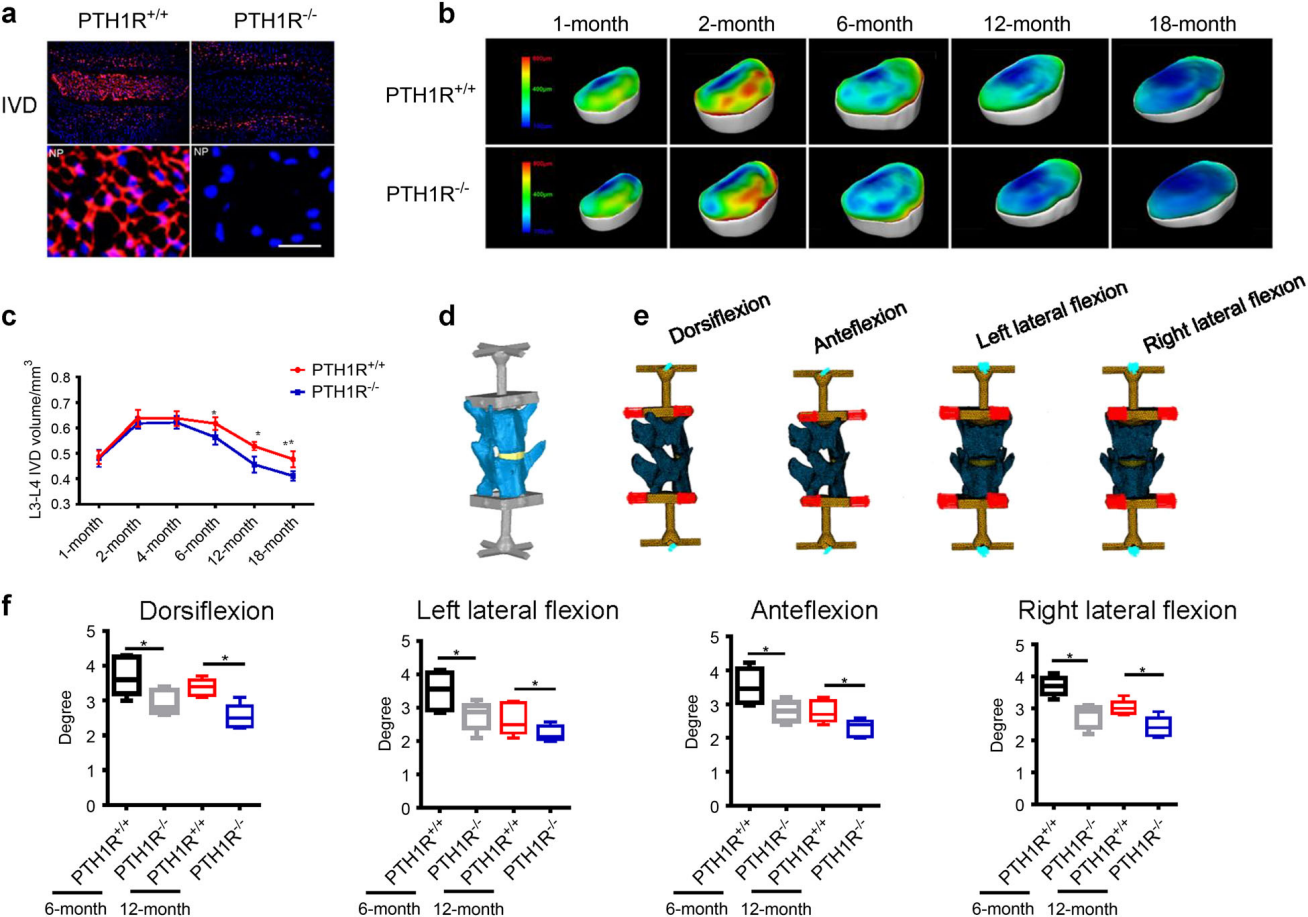
Conditional knockout of PTH1R reduces spinal flexibility. **a** Immunostaining images showing no PTH1R expression in NP tissue of PTH1R-deficient mice (PTH1R^−/−^) relative to their wild type littermates (PTH1R^+/+^). IVDs (top); NP (bottom). Scale bar, 50 μm. **b** 3D PPCT images of IVD thickness distribution in PTH1R^−/−^ mice at different ages compared to PTH1R^+/+^ mice. Scale bar, color code indicating the degree of thickness from blue (100 μm) to red (800 μm). **c** Quantitative analysis of IVD volume in PTH1R^−/−^ mice at different ages compared to PTH1R^+/+^ mice. **d**, **e** Images of the 3D finite element analysis model for testing spine flexibility in PTH1R-deficient mice at 6-month and 12-month-old mice. **d** The upper surface of L3 and bottom surface of L4 were fixed with rigid bars to mimic the loading of IVD for flexibility measurement. **e** For each model, the torque loading was applied to simulate motion in four different directions; dorsiflexion, anteflexion, left, and right lateral flexion measurement. **f** Quantitative analysis of spine flexibility in PTH1R-deficient mice at 6-month and 12-month of age. *P < 0.05, **P < 0.01. *n* = 8 per group. Statistical significance was determined by one-way ANOVA and Student's *t*-test. All data are reported as the mean ± s.d.

**Fig. 6:**
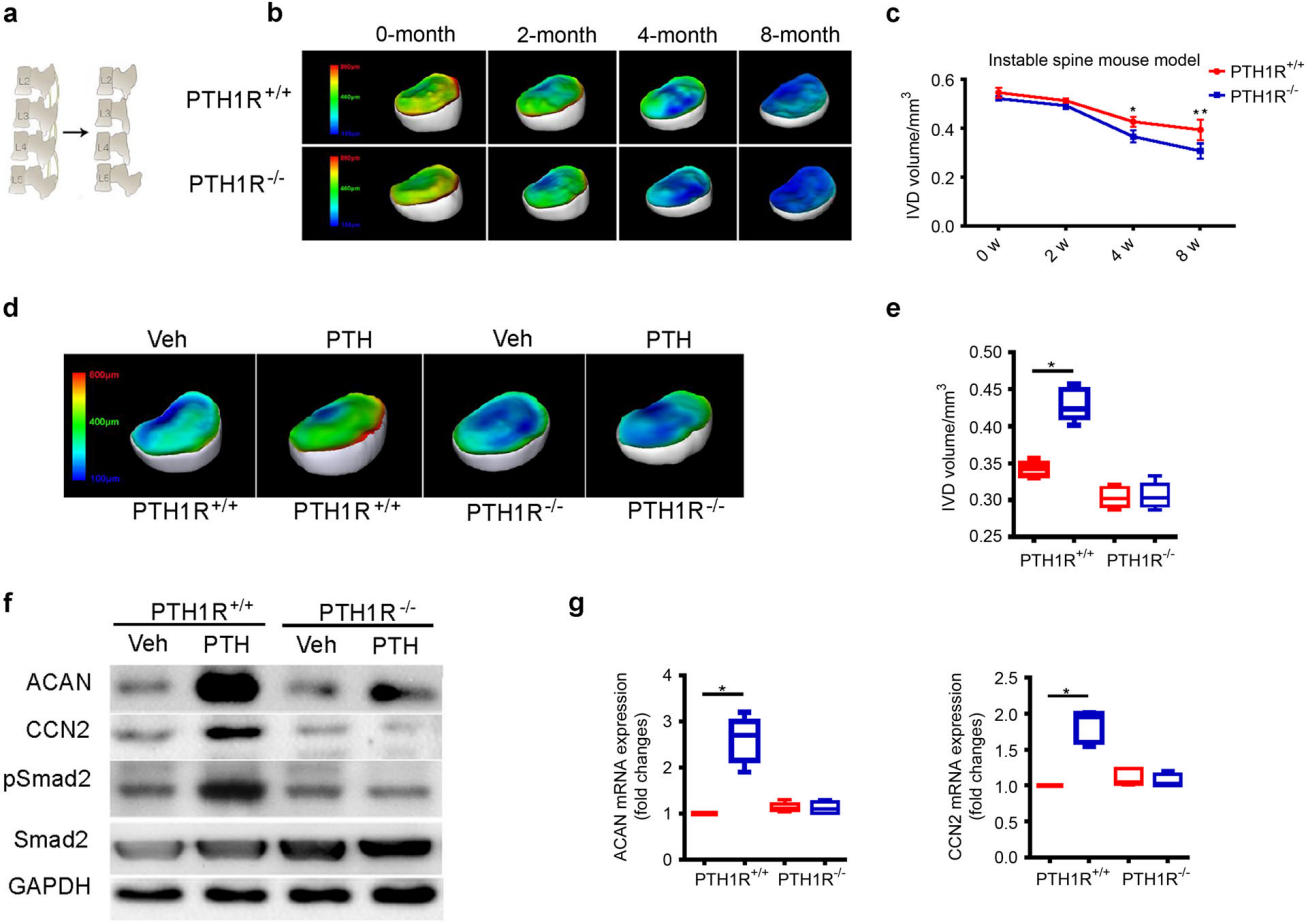
Conditional knockout of PTH1R accelerates disc degeneration. **a** Schematic representation of the unstable spine model generated by resection of the third and fourth lumbar spinous processes along with the supraspinous and interspinous ligaments from second to fifth lumbar vertebra. **b** 3D PPCT images of IVD thickness distribution in PTH1R-deficient mice at different time points post-surgery compared with those of PTH1R^+/+^ mice. **c** Quantitative analysis of IVD volume of (**b**). **d** 3D PPCT images showing IVD thickness distribution in 12-month-old PTH1R-deficient mice or PTH1R^+/+^ mice treated with iPTH or vehicle. **e** Quantitative analysis of IVD volume in (**d**). **f** Western blot analysis of pSmad2/3, CCN2 and Acan in NP tissue from PTH1R-deficient mice treated with iPTH or vehicle. **g** qRT-PCR analysis of the mRNA expression levels of CCN2 and Acan in NP tissue from PTH1R-deficient mice or PTH1R^+/+^ mice treated with iPTH or vehicle. Results reported as fold change. *P < 0.05, **P < 0.01. *n* = 8 per group. Statistical significance was determined by one-way ANOVA and Student's *t*-test. All data are reported as the mean ± s.d.

To investigate the role of PTH signaling in NP cells during aging, we crossed Noto^Cre^ mice with PTH1R^flox/flox^ mice to delete PTH1R gene specifically in notochord-derived NP cells (Noto^Cre^::PTH1R^flox/flox^, named “PTH1R KO mice” thereafter). PTH1R expression was undetectable in the NP cells in disc sections of PTH1R KO mice (Fig. 5a). The intervertebral disc changes were evaluated with PPCT in PTH1R KO mice and in their age-matched wild-type littermates (herein called PTH1R^+/+^ mice) at different ages. We found that intervertebral disc volumes decreased starting at 6-months of age relative to PTH1R^+/+^ mice, whereas postnatal intervertebral disc volumes and those in adulthood remained normal in PTH1R KO mice (Fig. 5b, c), suggesting a critical role of PTH in maintaining function of intervertebral disc during aging. In human, higher levels of PTH1R expression were also observed only in aged intervertebral discs (Fig. 2h). To assess the intervertebral disc function, the 3D finite-element analysis (FEA) model based on PPCT image acquisition was used for intervertebral disc flexibility testing, including dorsiflexion, ante-flexion, left, and right lateral flexion (Fig. 5d, e). Disc flexibility in all directions showed significant progressive decreases in PTH1R KO mice beginning at 6 months of age (Fig. 5f). Moreover, we generated a spine destabilized mouse model by resection of the intervertebral ligament of adjoining lumbar vertebrae to assess whether PTH regulates the function of discs under unstable mechanical loading environment (Fig. 6a). Intervertebral disc volume was decreased in 2-month-old PTH1R^−/−^ mice 4 weeks post-surgery, which was much earlier than that in the stable model where significant decrease started at 6-months of age and is much pronounced at 12-months (Fig. 6b, c).

We then examined whether the deletion of PTH1R in NP cells could influence the iPTH anabolic effects on the intervertebral disc. The increase in intervertebral disc volume caused by iPTH in 12-month-old PTH1R^+/+^ mice was abolished in PTH1R KO mice (Fig. 6d, e). Furthermore, Western blot analysis showed that the PTH-induced increase in levels of pSmad2, ACAN, and CCN2 seen in NP tissue was diminished in the PTH1R KO mice (Fig. 6f). PTH significantly increased the mRNA expression of ACAN and CCN2 in NP cells of wild-type mice by qRT-PCR, which were blunted in PTH1R KO mice (Fig. 6g). As disc volumes decreased with the decrease of proteoglycan starting at 6 months of age in PTH1R KO mice, this was likely an early stage of degeneration of accelerated sign of aging. Thus, PTH signaling in NP cells is essential for disc homeostasis, especially during aging.

### PTH stimulates transport of PTH1R to cilia of NP cells

**Fig. 7:**
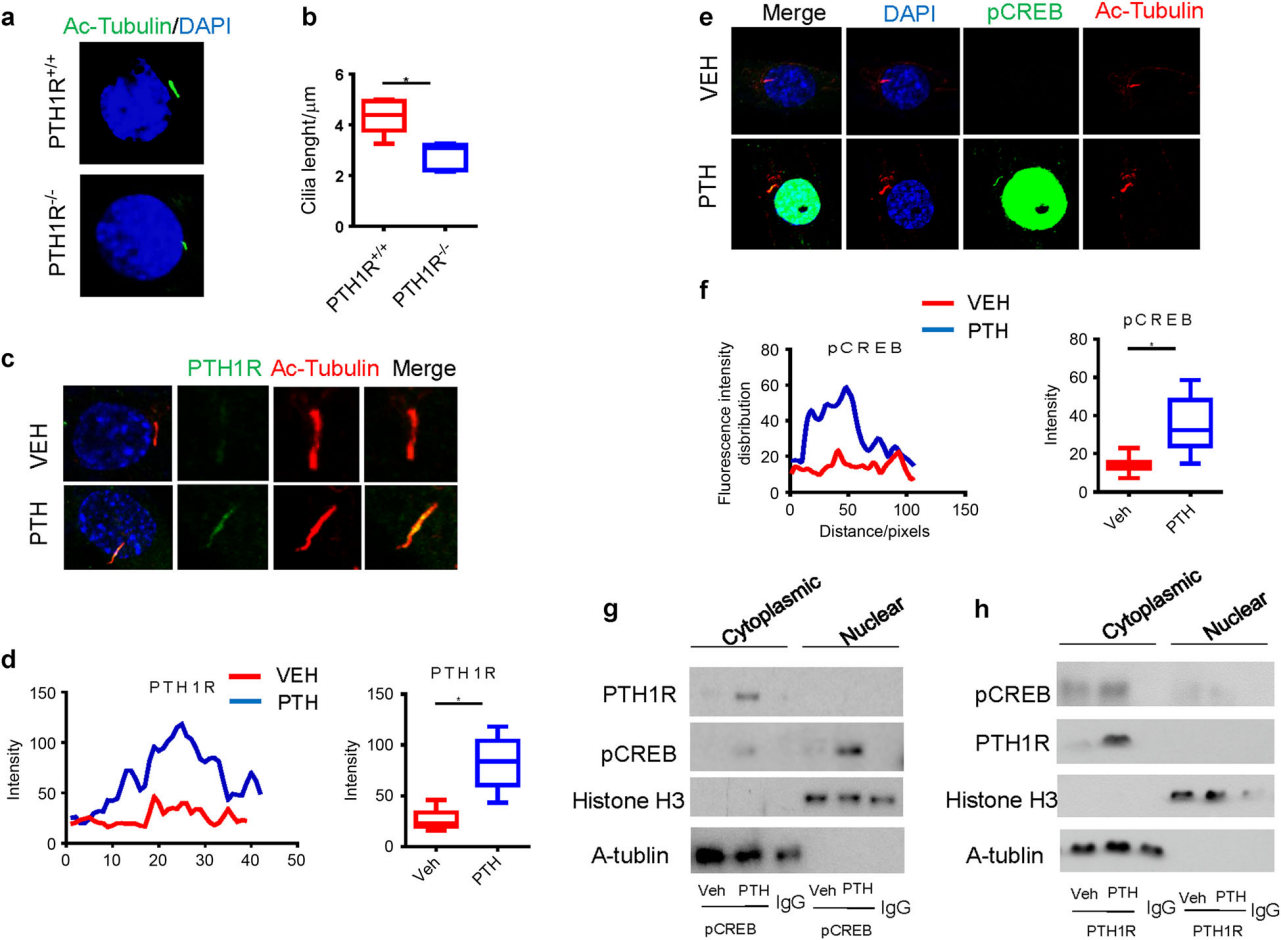
PTH stimulates transport of PTH1R to primary cilia of NP cells. **a** Immunostaining for acetylated α-tubulin (green) and DAPI (blue) showing the length of primary cilia of NP cells from PTH1R-deficient mice or PTH1R^+/+^ mice. **b** Quantitative measurements of primary cilia length of (**a**). **c** Immunostaining for acetylated α-tubulin or PTH1R showing that PTH-stimulated transport of PTH1R to primary cilia of NP cells. **d** Quantitative analysis of PTH1R intensity in cilia of (**c**). **e** Immunostaining for DAPI, pCREB, or acetylated α-tubulin showing that PTH-stimulated phosphorylation of CREB at primary cilia of NP cells. **f** Quantitative analysis of pCREB intensity in cilia of (**e**). **g**, **h** Co-immunoprecipitation of cell lysates from NP cells treated with PTH or vehicle using antibody against pCREB and blotted with PTH1R (**g**) or using antibody against PTH1R and blotted with pCREB (**h**) showing the interaction between PTH1R and pCREB in the acetylated α-tubulin extracts. *P < 0.05. *n* = 8 per group. Statistical significance was determined by one-way ANOVA and Student's *t*-test. All data are reported as the mean ± s.d.

To understand if mechanical stress regulates PTH signaling in NP cells, we investigated whether primary cilium in the NP cells regulates PTH signaling as PTH1R is a GPCR and found in primary cilia. Immunostaining of acetylated tubulin demonstrated that primary cilia were present in the NP cells and the length of primary cilia was significantly shorter in PTH1R KO mice (Fig. 7a, b). Importantly, the translocation of PTH1R to cilia was stimulated beginning at 10 min after administration of PTH (Fig. 7c, d). Co-immunostaining of pCREB with acetylated tubulin also revealed that pCREB increased in cilia (Fig. 7e, f), suggesting the effect of cilia on regulating PTH signaling. We then examined whether PTH1R and pCREB form a complex. Co-immunoprecipitation with an antibody against pCREB, blotted with PTH1R, demonstrated that the interaction between PTH1R and pCREB was only in the cytoplasmic acetyl tubulin, including cilia (Fig. 7g). Similarly, co-immunoprecipitation with antibody against PTH1R showed the interaction only present in the cytoplasmic acetyl tubulin (Fig. 7h).

**Fig. 8:**
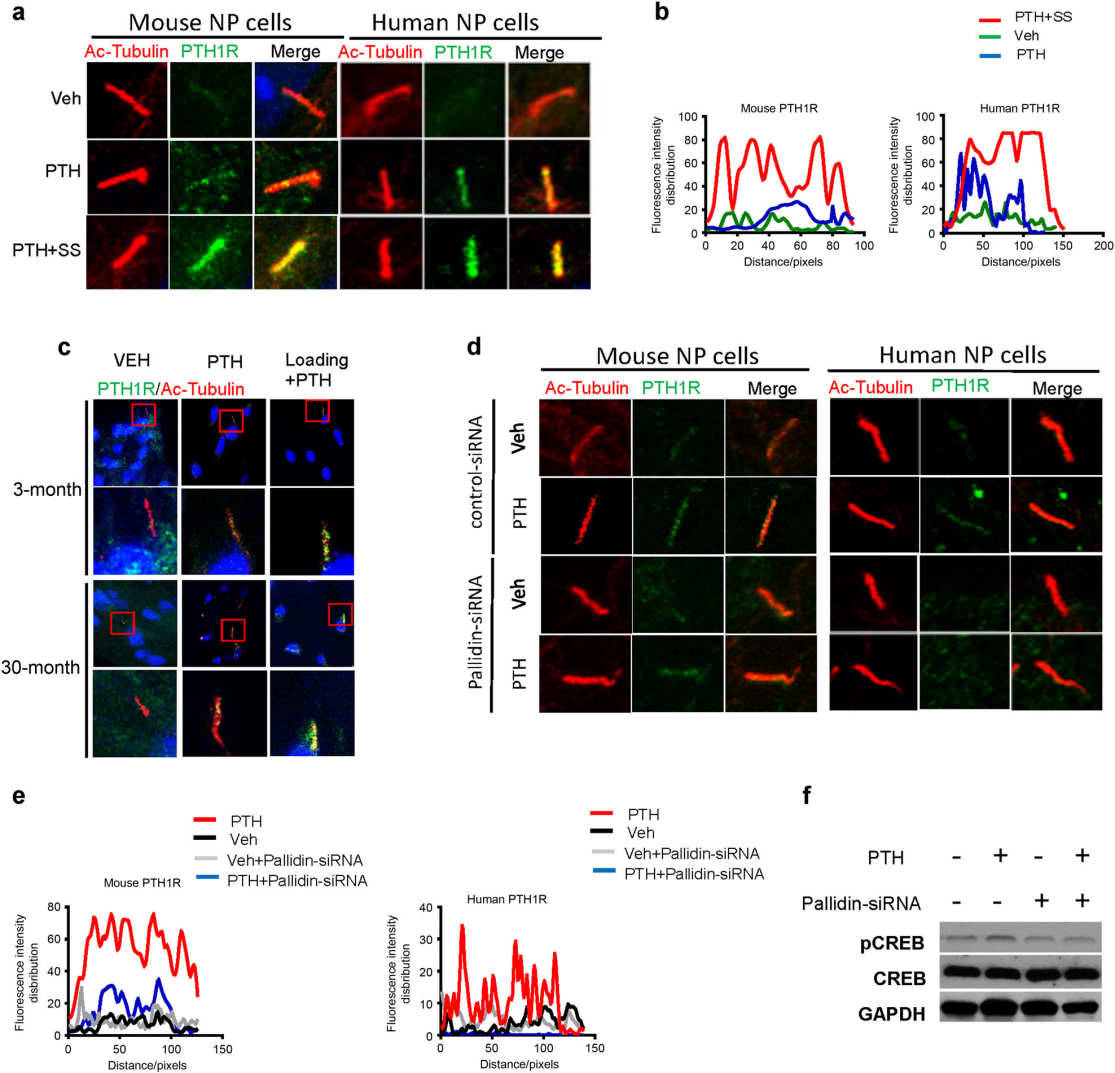
Shear stress enhances transport of PTH1R to primary cilia. **a** Immunostaining for acetylated α-tubulin or PTH1R showing that shear stress stimulated transport of PTH1R to primary cilia of both mouse and human NP cells. **b** Quantitative analysis of PTH1R intensity in mouse (left) and human (right) cilia of (**a**). **c** Immunostaining for acetylated α-tubulin or PTH1R showing that PTH-stimulated transport of PTH1R to primary cilia of NP cells in the presence of compression loading in an IVD ex vivo compression model of 3-month-old and 30-month-old rat. **d** Immunostaining of the mouse and human NP cells treated with pallidin siRNA or control siRNA for acetylated α-tubulin or PTH1R showing that PTH-stimulated transport of PTH1R to primary cilia of NP cells was inhibited. Scale bar, 10 μm. **e** Quantitative analysis of PTH1R intensity in mouse (left) and human (right) cilia of (**d**). **f** Western blot analysis of PTH-induced pCREB levels in NP cells treated with pallidin siRNA or control siRNA. *n* = 8 per group

We then examined whether mechanical stress regulates PTH signaling through cilia. PTH-induced translocation of PTH1R to cilia was significantly enhanced by shear stress in both mouse and human NP cells (Fig. 8a, b). Similar results were observed in the NP cells in ex vivo intervertebral disc organ culture of both young and aged rats with compressive mechanical loads. PTH-stimulated translocation of PTH1R to cilia and mechanical loads enhanced the translocation (Fig. 8c). Pallidin mediates the transport of proteins to cilia. We, therefore, knocked down pallidin with the small interfering ribonucleic acid (siRNA) to validate PTH-enhanced translocation of PTH1R to cilia. The knockdown of pallidin blocked PTH-enhanced transport of PTH1R to cilia in both mouse and human NP cells (Fig. 8d, e) and pCREB validated in Western blot analysis (Fig. 8f). Taken together, PTH stimulates transport of PTH1R to primary cilia and shear stress enhances the translocation.

### Disruption of cilia decreases intervertebral disc volume, PTH signaling and TGF-β activity in NP cells

**Fig. 9:**
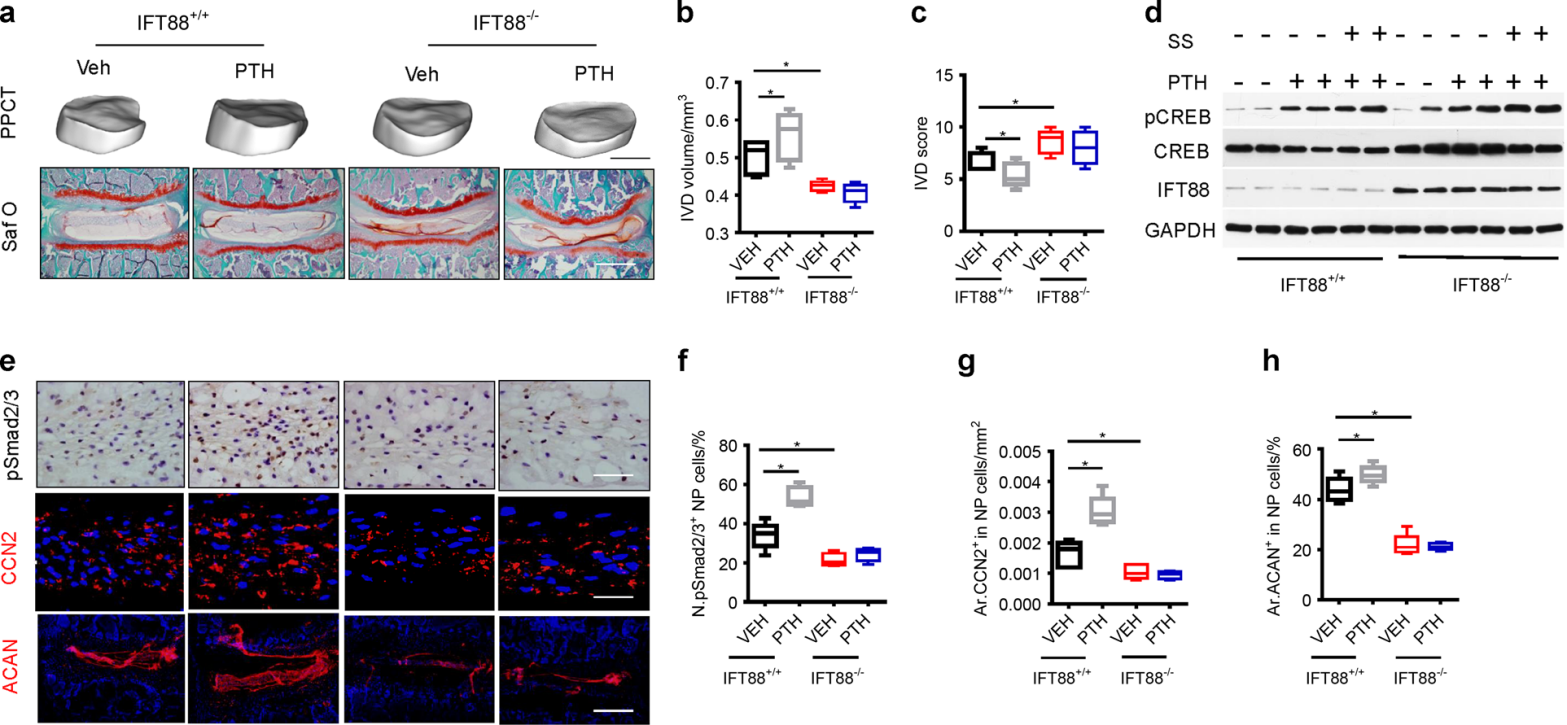
Primary cilia regulates PTH signaling in NP cells for disc anabolic activity. **a** 3D PPCT and immunostaining images showing that PTH effect on IVDs diminished in IFT88^−/−^ mice. PPCT Scale bar, 500 μm. Safranin-O Scale bars, 100 μm. **b** Quantitative analysis of IVD volume and (**c**) IVD histological scores of (**a**). **d** Western blot analysis of pCREB in NP cells isolated from IFT88^−/−^ or IFT88^+/+^ mice injected with iPTH or vehicle with or without shear stress. **e** Immunostaining for pSmad2/3 (brown), CCN2 and ACAN (red) with DAPI (blue) in 2-month IFT88^−/−^ or IFT88^+/+^ mice treated with PTH or vehicle. pSmad2/3 and CCN2 Scale bar, 50 μm; ACAN Scale bar, 100 μm. **f**, **g** and **h** Quantitative analysis of percentages of pSmad2/3 positive cells in NP area and CCN2 and ACAN-positive area of total IVD area. *P < 0.05. *n* = 8, per group. Statistical significance was determined by one-way ANOVA and Student's *t*-test. All data are reported as the mean ± s.d.

To examine the role of cilia in maintaining intervertebral disc function, we crossed IFT88^flox/flox^ mice with Noto^Cre/+^ mice to generate IFT88::Noto^Cre^ mice (IFT88^−/−^) to disrupt the cilia function specifically in NP cells. The intervertebral disc volume was significantly decreased in IFT88^−/−^ mice relative to their wild-type mice (Fig. 9a, b), and the intervertebral disc scores were increased consequently (Fig. 9a, c). Furthermore, iPTH-induced increase in intervertebral disc volume was blunted in IFT88^−/−^ mice relative to their wild-type mice (Fig. 9a, b), and the improvement of intervertebral disc scores by iPTH was also impaired (Fig. 9a, c). To validate that mechanical stress regulates PTH signaling through translocation of PTH1R to cilia, we examined whether PTH stimulates CREB phosphorylation in NP cells with disruption of primary cilia. Western blot analysis showed that PTH significantly stimulated the pCREB and shear stress enhanced the phosphorylation in wild-type mice (Fig. 9d). However, the disruption of primary cilia significantly reduced the levels of CREB phosphorylation by either PTH or shear stress in IFT88^−/−^ NP cells. Importantly, immunohistochemical staining demonstrated that PTH significantly stimulated the number of pSmad2/3^+^ NP cells and the levels of CCN2 and aggrecan in wild-type mice relative to mice with vehicle. The effect of PTH was blunted by disrupting of cilia specifically in NP cells in IFT88^−/−^ mice (Fig. 9e–h). These data demonstrate that mechanical stress enhances PTH signaling in NP cells via primary cilia to maintain intervertebral disc function.

## Discussion

There is no disease-modifying therapy for DDD due to the unclear pathogenesis of this disease. The current management modalities including total disc replacement and spinal lumbar fusion are unable to effectively slow or reverse the disease process. Spine provides the stable mechanical support for the body and protection of spinal cord with an array of joined vertebral bodies with cartilage at each end. Intervertebral discs, gel-like soft tissue, connect the vertebral bodies to provide flexibility for spine and absorb the mechanical loading transmitted along the spinal axis. In this study, we found that mechanical stress induces transport of PTH1R to primary cilia of NP cells to stimulate PTH-induced expression of β_6_ integrin in activation of extracellular latent TGF-β. Importantly, mechanical loads in discs are necessary for transport of PTH1R to cilia in activation of PTH downstream signaling. iPTH treatment attenuates disc degeneration during aging, and disruption of primary cilia specifically in NP cells blunted the effect. Moreover, PTH1R was highly expressed in NP cells of aged rat and human discs (Fig. 2; Fig. 8a–d). Knockout of PTH1R postnatally in NP cells has little effect on intervertebral discs, however, resulted in accelerated disc degeneration of adult mice. Thus, primary cilia in NP cells regulate PTH signaling in response to mechanical loading to maintain intervertebral disc homeostasis, particularly during aging.

The parathyroid gland evolved in amphibians as the primary site for PTH production.^40^ PTH regulates bone remodeling and calcium homeostasis in adaptation to terrestrial life as the transition from aquatic vertebrates.^41–44^ The discs of terrestrial vertebral animals bear mechanical loads different from that of aquatic animals. The fact that PTH signaling components exist in NP cells further suggests that PTH evolved as a hormone during the transition for adaptation to terrestrial locomotion. The evolution of humans began in primates only about four million years ago,^45^ whereas the PTH gland evolved in amphibians about 370 million years ago.^40^ Our findings suggested that PTH-signaling pathway in NP cells is well preserved in human bipedalism. Furtherance to the cloning of the gene encoding the PTH receptor in 1991, significant strides have been made in understanding the physiology of PTH, as well as testing its molecular and cellular functions. In addition to its direct downstream signaling, we have demonstrated that PTH has obtained the ability in adaptation to terrestrial locomotion by the orchestration of signaling of local factors including TGF-β, Wnts, BMP, and IGF-1^30–34^ to maintain skeletal homeostasis. A number of recent studies have implicated PTH activity in spine function. Supplementation of PTH has been proposed to suppress calcification markers in human intervertebral disc cells.^46^ PTH helps in retarding intervertebral disc degeneration by significantly decreasing calcification in discs through both MAPK and PKA signaling pathways.^46^ iPTH injection improves the adjacent segment disc degeneration by enhancing lumber fusion^47^ and alleviates disc degeneration in ovariectomized osteoporotic mice by up-regulation of Wnt/β-catenin pathway.^48^ In this study, we demonstrate that PTH1R is expressed in the NP cells of notochordal origin to maintain disc homeostasis particularly during aging by directly activating TGF-β signaling in NP cells in addition to its expression in vertebral bodies and endplates. The results suggested that expression of PTH1R in disc cells promotes the mobility of amphibians in their adaptation to terrestrial life from aquatic vertebrates.

The NP, of notochord origin represents a centrally located gelatinous homogenous mass consisting of cells surrounded by ECM.^49,50^ The ECM is a highly hydrated tissue made up of proteoglycan and collagen. The predominant types of proteoglycan and collagen are aggrecan and collagen type II, respectively. Functionally, the collagen imparts tensile strength, while the proteoglycans attract and bind water, providing resilience to compression. The ECM is central to the integrity and function of the NP and hence intervertebral disc. Loss of ECM content especially in the NP is associated with disc degeneration.^51^ Antoniou et al. demonstrated age-loss of glycosaminoglycans of aggrecan specifically occurs in the NP.^52^ We found the decreases in proteoglycan and collagen II in the NP with aging were improved after treatment with iPTH. The role of TGF-β in ECM homeostasis was well studied in disc degeneration. TGF-β induces CCN2 expression to increase ECM synthesis in the cartilage and discs.^53^ Transgenic mice carrying a dominant-negative TGF-β type II receptor develop spinal abnormalities;^54^ knockout of Smad3, a TGF-β downstream signaling factor, in mice also results in spinal deformity, caused by loss of cartilage and the decreased production of proteoglycans.^55^ Furthermore, TGF-β1 treatment decreases the expression of matrix metalloproteinases that are involved in tissue remodeling events in disc degeneration.^55^ We found that integrin α_v_β_6_ mediates mechanical stress by activation of latent TGF-β, acting as a cellular mechanotransductor for disc physiological function.^18^ PTH induces expression of integrin α_v_β_6_ to regulate mechanical stress-induced activation of latent TGF-β. Moreover, primary cilia enhance PTH signaling to induce integrin α_v_β_6_ expression in activation of TGF-β in intervertebral discs. Treatment of aged mice with iPTH resulted in increased active TGF-β levels and concomitant increase in aggrecan and collagen II, which confirmed the role of TGF-β in the PTH-mediated maintenance of disc homeostasis.

DDD is managed palliatively with pain medications and life-style modification to alleviate pain and reduce functional impairment. When pain becomes disabling, surgery may be performed, such as spinal fusion. Our study demonstrates that PTH-induced activation of TGF-β to maintain disc homeostasis in animal models with the implication of potential modification of the disease. As we know, PTH1R is also expressed in chondrocytes of endplates, osteoblasts, and osteocytes in vertebral bodies, and iPTH also has the effect on both endplates and vertebral bodies. Discs depends on nutrients, oxygen, etc. from diffusion of sounding tissue with blood vessels, particularly endplates. During aging or disc degeneration, endplates become calcified and sclerotic, leading to less efficient diffusion of nutrients in support for discs.^56,57^ iPTH will stimulate osteoclast resorption of the sclerotic endplates and induce native bone formation through remodeling,^48^ and the process likely improves their diffusion property for the disc. Similarly, iPTH stimulates bone remodeling and improves bone quality for the vertebral bodies. Therefore, iPTH has an effect on disc homeostasis by acting on endplates and vertebral bodies in addition to direct signaling through NP cells. In human discs, NP cell density is low and during aging endplates are severely sclerosis. iPTH could also induce remodeling of sclerotic endplates to improve diffusion in attenuation of disc degeneration in human.

## Methods

### Human subjects

After approval by the Institutional Review Board of Johns Hopkins University, we collected lumbar disc specimens from five patients (14-year, 15-year, 24-year, 68-year, 72-year-old, respectively). The status of disc degeneration of the specimens was examined with the Pfirrmann grading system based on T2-weighted magnetic resonance imaging. All the discs of these five patients with different age were in grade III who had received posterior discectomy and fusion surgeries. The AF tissue cut by sharp-pointed knives during fenestration discectomy. NP tissue was taken from the inner part of IVD, which was gelatinous. Different disc regions were cleanly separated from each other and clearly distinguished for unambiguous dissection. The specimens were processed for Western blot examination.

### Mice

For ageing-induced intervertebral disc degeneration model, 2 and 16 months-old C57BL/6J WT male mice were purchased from Charles River Laboratories (Wilmington, MA). We purchased Gt (ROSA) 26Sor^tm1Sor^/J mice from the Jackson Laboratory (Bar Harbor, ME). Mice with floxed PTH1R (PTH1R^flox/flox^) were obtained from the lab of Dr. Henry Kronenberg.^58^ We obtained the Noto^Cre^ mice strain from Dr. Cheryle A. Seguin.^8,17^ Mice carrying the *PTH1R* gene flanked by loxP sites (PTH1R^flox/flox^) were mated with Noto^Cre^ mice^17^ to generate mice bearing Noto^Cre^ and a floxed PTH1R allele in their germline. These mice were backcrossed to homozygous floxed mice (Noto^Cre/+^/ PTH1R^flox/+^:: PTH1R^flox/flox^) to generate mice with inactivation of both alleles in notochord-derived cells (genotype Noto^Cre/+^:: PTH1R^flox/flox^). Homozygous disruption of the Noto locus is perinatally lethal;^59^ viable offspring have genotypes of either Noto^Cre/+^:: PTH1R^flox/flox^ (mice with PTH1R conditional deletion in Noto lineage cells are referred to as “PTH1R^−/−^” in the text) or Noto^+/+^:: PTH1R^flox/flox^ (wild-type littermates hereinafter referred to as “PTH1R ^+/+^” in the text). The genotype of transgenic mice was determined by PCR analyses of genomic DNA isolated from mouse tails. The floxed PTH1R allele was identified with primers lox1F (5′–TGGACGCAGACGATGTCTTTACCA–3′) and lox1R (5′–ACATGGCCATGCCTGGGTCTGAGA–3′). The genotyping for the Cre transgene was performed by PCR with the primers Cre F (5′–CAAATAGCCCTGGCAGAT–3′) and Cre R (5′–TGATACAAGGGACATCTTCC–3′). We generated Noto^Cre/+^:: ROSA26-lacZ^flox/flox^ by crossing Noto^Cre^ mice with mice homozygous with a loxP-flanked DNA stop sequence, preventing expression of the downstream *lacZ* gene. IFT88 ^flox/flox^ mouse model has been generated as descripted. A NP tissue-specific primary cilia knock-out (KO) mouse line was generated by crossing Noto^Cre^ mice with IFT88 ^flox/flox^ mice, in which the primary cilia were deleted from the NP tissue. The loxP IFT88 allele was identified with the primers lox1F (5′–GACCACCTTTTTAGCCTCCTG–3′) and lox1R (5′–AGGGAAGGGACTTAGGAATGA–3′).

For lumbar spine instability mouse model (LSI): 2 months old PTH1R^+/+^ and PTH1R^−/−^ male mice were used for this experiment. After anesthetizing with ketamine and xylazine, mice were operated by resection of the Lumbar^3rd^–Lumbar^4th^ (L3–L4) spinous processes along with the supraspinous and interspinous ligaments to induce instability of lumbar spine. Mice were euthanized at 0, 2, 4, and 8 weeks after the surgery (*n* = 8 per group).^18,56^

For the dosage screening experiments, 17 months old male C57BL/6J WT mice were assigned into four groups treated with different doses of PTH or vehicle; 20, 40, and 80 μg·kg^-1^ per day of human PTH (1–34) (Bachem, Inc., King of Prussia, PA, USA) or vehicle groups. We chose the optimal dosage of PTH (1–34) for the rest of the experiment.

The 11 months olds PTH1R^+/+^ and PTH1R^−/−^ male mice were randomized into four groups: (1) PTH1R^+/+^ + PTH (1–34); (2) PTH1R^+/+^ plus vehicle; (3) PTH1R^−/−^ plus PTH (1–34); and (4) PTH1R^−/−^ plus vehicle (*n* = 8 per group). The 11-month-old IFT88^+/+^ and IFT88^−/−^ male mice were randomized into four groups: (1) IFT88^+/+^ plus PTH (1–34); (2) IFT88^+/+^ plus vehicle; (3) IFT88^−/−^ plus PTH (1–34); and (4) IFT88^−/−^ plus vehicle (*n* = 8 per group). Mice were subcutaneously injected with either PTH (1–34) or vehicle (1 mmol·L^-1^ acetic acid in phosphate buffered saline (PBS) with equivalent volume of PTH) daily, 5 days per week, and all mice were sacrificed 4 weeks after treatment with PTH (1–34) or vehicle.

All animals were maintained in the Animal Facility of the Johns Hopkins University School of Medicine. The experimental protocols for both species were reviewed and approved by the Institutional Animal Care and Use Committee of The Johns Hopkins University, Baltimore, MD, USA.

### IVD ex vivo compression model

The L1–L5 lumbar IVDs of 3-month-old and 30-month-old Sprague Dawley rat were removed under sterile conditions. The collected IVDs were organ cultured in Dulbecco’s modified Eagle’s medium (DMEM) (Invitrogen, Carlsbad, CA, USA) supplemented with 1% penicillin–streptomycin (MediaTech, Dallas, TX, USA) and treated with either PTH (100  nmol) or vehicle. Meanwhile, static compression loads of 0 or 1.0 MPa were applied on the IVDs by vertically placing weights. Treatment duration was 3 or 48 h (*n* = 6 per group).

### Primary NP cell isolation and culture

GFP-labeled NP cells of notochordal origin were isolated from 15-day-old Noto^Cre/+^::ROSA26-lacZ^flox/flox^ male mice as previously described^60.^ The NP cells from 15-day-old PTH1R^+/+^ and PTH1R^−/−^, IFT88^+/+^ and IFT88^−/−^ male mice. Briefly, the cells were isolated from the NP region of intervertebral discs in the spinal column from mid thoracic to lower lumbar region and digested initially with TrypLE Express (Gibco Laboratories, Gaithersburg, MD) for 30 min on shaker, followed by 0.25 mg·mL^-1^ Collagense-P (Roche) for another 30 h at 37 °C. The digested cells were washed twice with PBS and cultured in α-MEM (Gibco) supplemented with 10% fetal calf serum (Atlanta Biologicals, Flowery Branch, GA), and 1% penicillin–streptomycin (MediaTech, Dallas, TX, USA) to 80%–90% confluence at 37 °C, 5% CO_2_, and 5% O_2_.

Human NP cells were isolated from disc tissues removed during surgical intervention for spine fracture in two young patients. Tissue specimens were placed in sterilized Ham’s F-12 medium supplemented with 10% fetal bovine serum (FBS; Gibco-BRL) and 1% penicillin/streptomycin and the disc tissues were washed twice with Hank’s balanced salt solution (HBSS; Gibco-BRL) to remove bodily contaminants before isolation. An incision was then made in the NP part of the disc. The NP tissue were digested for 60 min in F-12 medium containing 1% penicillin–streptomycin, 10% FBS, and 0.2% pronase (Calbiochem, La Jolla, CA, USA), followed by incubation in medium with 0.025% collagenase for 24 h. A sterile mesh filter (70 μm pore size) was used to remove tissue debris, and the NP cells were isolated. The supernatant was centrifuged at 700×*g* for 5 min and resuspended in F-12 medium containing 10% FBS and 1% penicillin–streptomycin. The cells were cultured in 75 cm^2^ cell culture flasks at 37 °C in a humidified atmosphere with 5% CO_2_.

### Primary cilia visualization and co-localization analysis

The localization of PTH signaling components within NP cells was investigated through immunocytochemistry after 48 h of serum starvation (DMEM, 0.5% FBS, 1% penicillin–streptomycin). Cells were treated with PTH (recombinant human PTH, 100 nmol, Bachem California, Inc., King of Prussia, PA, USA) or shear stress (1 dyn·cm^-^^2^) with times of exposure for 30 min, fixed and stained for acetylated α-tubulin of primary cilia, PTH1R (Abcam, 1:100) and pCREB (Abcam, 1:500). Coverslips were mounted with fluoroshield-DAPI. All cells were imaged using a Zeiss LSM710 META Confocal Laser Scanning microscope fitted with a 63× objective lens. Region of interest were selected manually using ImageJ software. The intensity profiles along the cilia have been determined by tracing a line across the length of the primary cilia and measuring intensity along this line using ImageJ software. Average intensities in the ciliary region, were measured on at least 30 ciliated cells per condition.

### Western blot analysis and co-immunoprecipitation

Western blot analyses were conducted on the protein lysates from in vitro-cultured NP cells or NP tissues from mice at specific time points after PTH (1–34) treatment and human at different ages with lumbar disc degeneration. The protein extract was centrifuged, the concentration of supernatant evaluated by detergent-compatible protein assay (Bio-Rad Laboratories, Hercules, CA), and then separated by sodium dodecyl sulfate polyacrylamide gel electrophoresis and blotted on a polyvinylidene fluoride membrane (Bio-Rad Laboratories). Following incubation in specific antibodies, we detected proteins using an enhanced chemiluminescence kit (Amersham Biosciences, Little Chalfont, United Kingdom). We used specific antibodies recognizing rabbit CTGF (CCN2) (Abcam, 1:1 000), Aggrecan (ACAN) (Abcam, 1:1 000), p-Smad2 (Cell Signaling Technology Inc., 1:1 000), Smad2 (Cell Signaling Technology Inc., 1:1 000), glyceraldehyde 3-phosphate dehydrogenase (GAPDH) (Cell Signaling Technology Inc., 1:1 000), CREB (Abcam, 1:2 500), pCREB (Abcam, 1:2 500), PTH1R PRB-635P (Covance, 1:100), PTH1R PRB-640P (Covance, 1:500), and goat integrin β_6_ (Santa Cruz, 1:500) to examine the protein concentrations in the lysates.

Co-immunoprecipitation analyses were conducted on the nuclear and cytoplasmic protein extraction using NE-PER Nuclear and Cytoplasmic Extraction Kit (Pierce Thermo Scientific, 78833) from in vitro-cultured NP cells treated with PTH (1–34) or vehicle. PTH1R and pCREB expression were revealed with an anti-pCREB (Abcam, 1:500) and anti-PTH1R (Abcam, 1:500) antibodies.

### ChIP assay

The ChIP assays were carried out using the Thermo Fisher ChIP Kit (catalog number: 26156). The crude homogenate from the NP cells was crosslinked with 1% formaldehyde at room temperature for 10 min. The reaction was stopped by adding glycine (0.25 mol·L^-1^). After centrifugation, the pellet was collected and lysed in sodium dodecyl sulfate lysis buffer containing protease inhibitor cocktail. The lysis was sonicated until the DNA was broken into fragments with a mean length of 200bps–1 000 bps. The samples were first pre-cleaned with protein G agarose and then subjected to immunoprecipitation overnight with 2 mg of rabbit antibodies against pCREB (CST, 1:50) overnight at 4 °C. The 10%–20% of the sample for immunoprecipitation was used as an input (a positive control). After purification, the DNA fragments were amplified using qRT-PCR with the primers for β6 promoter listed in Supplementary Table 1.

### cAMP assays and ELISA analysis

For cAMP assays, confluent cells were grown in 35-mm six-well plates starved overnight by incubation in serum-free α-MEM at 37 °C. The cells were then treated with 100 nmol·L^-1^ of human PTH (1–34; Bachem California, Inc.) for 1 h. Cellular cAMP was extracted and concentration measured with the Biotrak enzyme immunoassay system (GE Healthare, Inc., Princeton, HJ). We determined the concentration of total and active TGF-β in the NP tissue using the ELISA Development Kit (R&D Systems, Minneapolis, MN) according to the manufacturer’s instructions.

### Quantitative RT-PCR

After protein extraction, specimen from the same group of mice were prepared for RNA extraction using TRIzol reagent (Invitrogen, Carlsbad, CA) according to the manufacturer’s instruction. The purity of RNA was tested by measuring the absorbance at 260 nm and 280 nm. For qRT-PCR, two micrograms of RNA were reverse transcribed into complementary DNA using the SuperScript first-strand synthesis system (Invitrogen) and analyzed with SYBR Green-Master Mix (Qiagen, Hilden, Germany) in the thermal cycler with two sets of primers specific for each target gene. Relative expression was calculated for each gene by the 2^−△△CT^ method, with GAPDH for normalization. Primers used for qRT-PCR are listed in supplementary Table 2.

### Immunocytochemistry, immunofluorescence, and histomorphometry

For immunocytochemical staining, we incubated GFP-labeled NP cells co-stained with primary antibody to rabbit PTH1R PRB-635P (Covance, 1:100) for 1 h and subsequently stained with secondary antibodies conjugated with fluorescence at room temperature. At the time of euthanasia, we dissected and fixed the lumbar vertebral spine in 10% buffered formalin for 48 h, decalcified them in 10% ethylenediaminetetraacetic acid (EDTA) (pH 7.4) for 14–21 days and embedded them in paraffin or optimal cutting temperature (OCT) compound (Sakura Finetek, Torrance, CA). Four-micrometer-thick coronal-oriented sections of the L1–L6 spine were processed for safranin O and fast green staining. Sections for immunostaining were processed using a standard protocol and incubated with primary antibodies to rabbit ACAN (Abcam, 1:100), CCN2 (Abcam, 1:100), integrin β_8_ (Abcam, 1:200), pCREB (Abcam, 1:100), and PTH1R PRB-635P (Covance, 1:100), mouse pSmad2/3 (Santa Cruz, 1:100), integrin α_v_β_6_ (Millipore, 1:100), integrin α_v_β_3_ (Bioss, 1:100), integrin α_v_β_5_ (Bioss, 1:100) at 4 °C overnight. For immunohistochemical staining, a horse radish peroxidase–streptavidin detection system (Agilent, Santa Clara, CA) was subsequently used to detect the immunoactivity, followed by counterstaining with hematoxylin (Sigma-Aldrich, St. Louis, MO). For immunofluorescent assay, the slides were incubated with secondary antibodies conjugated with fluorescence at room temperature for 1 h while avoiding light. We used isotype-matched controls, such as polyclonal rabbit IgG (R&D Systems, AB-105-C) under the same concentrations and conditions as negative controls. We microphotographed sections to perform histomorphometric measurements on the entire area of the L3–L4 of the spine (Dp71 Microscope Camera, Olympus, Tokyo, Japan).

Quantitative histomorphometric analysis was conducted in a blinded fashion with Image-Pro Plus Software version 6.0 (Media Cybernetics Inc., Rockville, MD). intervertebral disc histological score were obtained as previously described.^61^ Five randomly selected sections per mice in each group at L3–L4 level were chosen for quantitative histomorphometric analysis. The percentage of pSmad2/3 and pCREB-positive cells was obtained by counting the number of positive staining cells to the number of total cells in the NP region. The percentage area of CCN2 and ACAN positive staining was calculated by measuring the positive area to the whole area of the L3–L4 in each group.

### PPCT scanning

PPCT based on the Synchrotron radiation scanning was performed at the BL13W1 biomedical beamline in the Shanghai Synchrotron Radiation Facility (SSRF). To obtain high X-ray attenuation contrast images, the monochromatic X-ray energy was adjusted to 15 keV, the scanner was set at a voltage of 15 keV, exposure time set to 2.5 s, and the sample-to-detector distance (SDD) adjusted to 30 cm and a resolution of 3.7 μm per pixel. The images were reconstructed and analyzed using the GPU-CT-Reconstruction software (applied by the BL13W1 experimental station) and the VG Studio Max 3D software (version 2.1, Volume Graphics GmbH, Germany) respectively. The region of interest was defined to cover the whole L3–L4 compartment. The 3D quantitative analysis of intervertebral discs was performed with the commercially available Image Pro Analyzer 3D software (Version 7.0, Media Cybernetics, Inc., USA). The 3D structural parameters were used to determine the mean height and volume and thickness distribution of intervertebral discs. The trabecular bone of L4 was segmented from the bone marrow and analyzed to determine the trabecular bone volume fraction (BV/TV), trabecular thickness (Tb. Th), trabecular number (Tb. N), and trabecular separation (Tb. Sp).

### PPCT-based 3D finite element test

For each model, PPCT images of one motion segment of L3–L4 were first subjected to noise elimination, and binarization was performed using thresholds obtained by discrimination analysis. Then 3D geometry models of bone tissue (including the cartilaginous endplate) and intervertebral disc were reconstructed from the segmentation results. For bone tissue model, because of the large amount of trabecular microstructure, use of the geometry solid model may result in topological errors and undesired geometric features; hence, the need to preprocess with geometry repair and optimization operations. Subsequently, the finite-element mesh models can be directly discretized by 10-node quadratic tetrahedral elements from the geometry solid models, by an adaptive meshing tool in the VG Studio Max 3D software. The material properties of tissues were set according to previous studies.^62–64^ Finally, FEA was carried out in ABAQUS (Dassault Systèmes Americas Corp, Waltham, MA, USA). For each model, the upper surface of L3 and bottom surface of L4 were fixed with rigid bars, such as the orange part shown in supplementary Figure 3b, to which torque loading can be applied precisely to simulate motion in four different directions; dorsiflexion, anteflexion, left, and right lateral flexion measurement. Finally, the resulting range of motion (ROM) of each motion segment was calculated.

### In vivo micro-MRI

In vivo Spinal intervertebral disc imaging was conducted with a horizontal 30-cm-bore 9.4 T Bruker Biospec preclinical scanner equipped with custom-built, single-turn volume coil positioned orthogonal to the B0 magnetic field. Anesthetization of mice was initiated with 4% isoflurane and maintained with a 2% isoflurane and oxygen mixture. Mice were placed supine on a tray and taped to minimize the motion artifacts. We acquired T2-weighted images using a rapid acquisition with relaxation enhancement (RARE) sequence with the following parameter: an echo time/repetition time (TE/TR) of 15.17 ms/3 000 ms, 35 slices at thickness of 0.35 mm, field of view (FOV) of 1.75 cm × 1.75 cm and matrix size of 256 × 128. All T2-weighted images were processed to a final matrix size of 256 × 256 with an isotropic resolution of 0.068 mm pixel-1. For quantification of signal intensity as an indicator of disc tissue hydration, the region of interest at L3–L4 level in each group was selected and measured using Image-Pro Plus Software version 6.

### Statistics

Data are presented as the mean ± s.d. We used unpaired, two-tailed Student’s *t*-tests for comparisons between two groups and one-way analysis of variance (ANOVA) with Bonferroni post hoc test for multiple comparisons. All data demonstrated a normal distribution and similar variation between groups. The level of significance was set at *P* < 0.05. All data analyses were performed using SPSS 22.0 analysis software (IBM, Armonk, NY).

## Electronic supplementary material

Supplementary Figures and tables
