# Supplementary material for: Ciliary parathyroid hormone signaling activates transforming growth factor-β to maintain intervertebral disc homeostasis during aging
Source: Bone Res. 2018 Jul 18;6:21. doi: 10.1038/s41413-018-0022-y (PMC6050246; doi:10.1038/s41413-018-0022-y)
Supplement: Supplementary file 1 — Supplementary Figures and tables [file 41413_2018_22_MOESM1_ESM.pdf]

Supplementary information

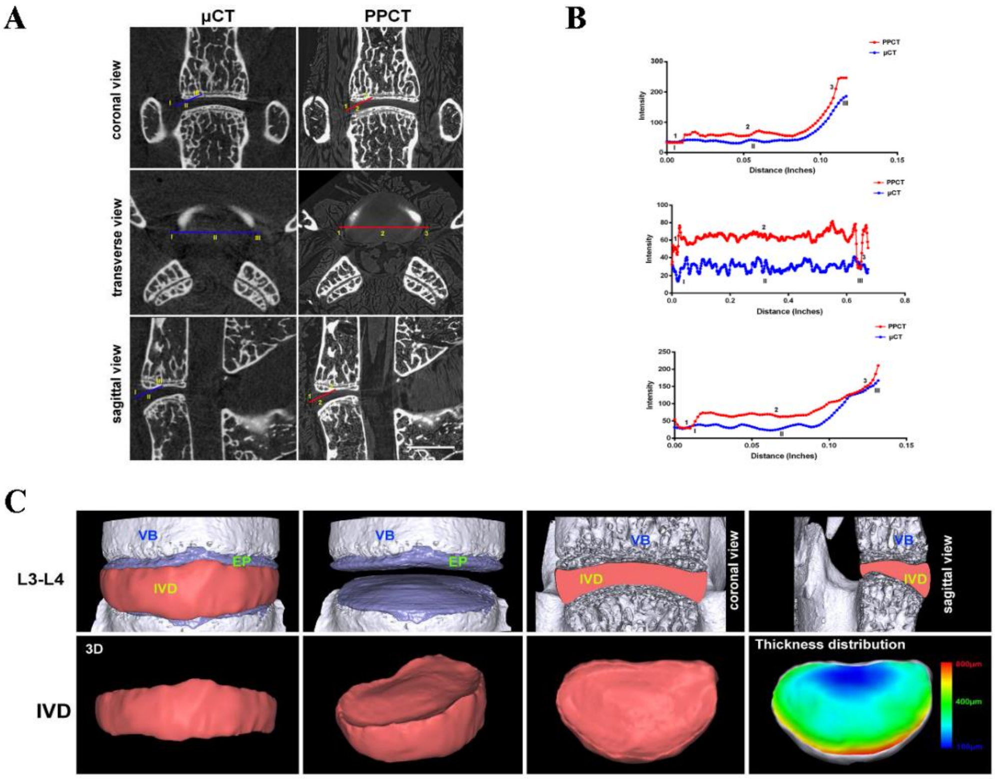

Supplementary Figure 1 3D visualization of IVDs by propagation phase contrast micro-tomography (PPCT) based on the Synchrotron radiation. (A) Coronal, transverse and sagittal views of the intervertebral disc were detected by PPCT; these views of the disc are difficult to delineate with conventional μCT. (B)The intensity distribution of the correspondence lines marked in (A). The blue line (I, II and III) refer to μCT, whereas the red line (1, 2 and 3) refer to PPCT. (C) Intact 3D images of the L3-4 segment. The vertebra (VB), endplate (EP) and IVD could be visualized separately and from multiple perspectives. The 3D thickness distribution could be defined using different color coding. Scale bar, 500 μm.

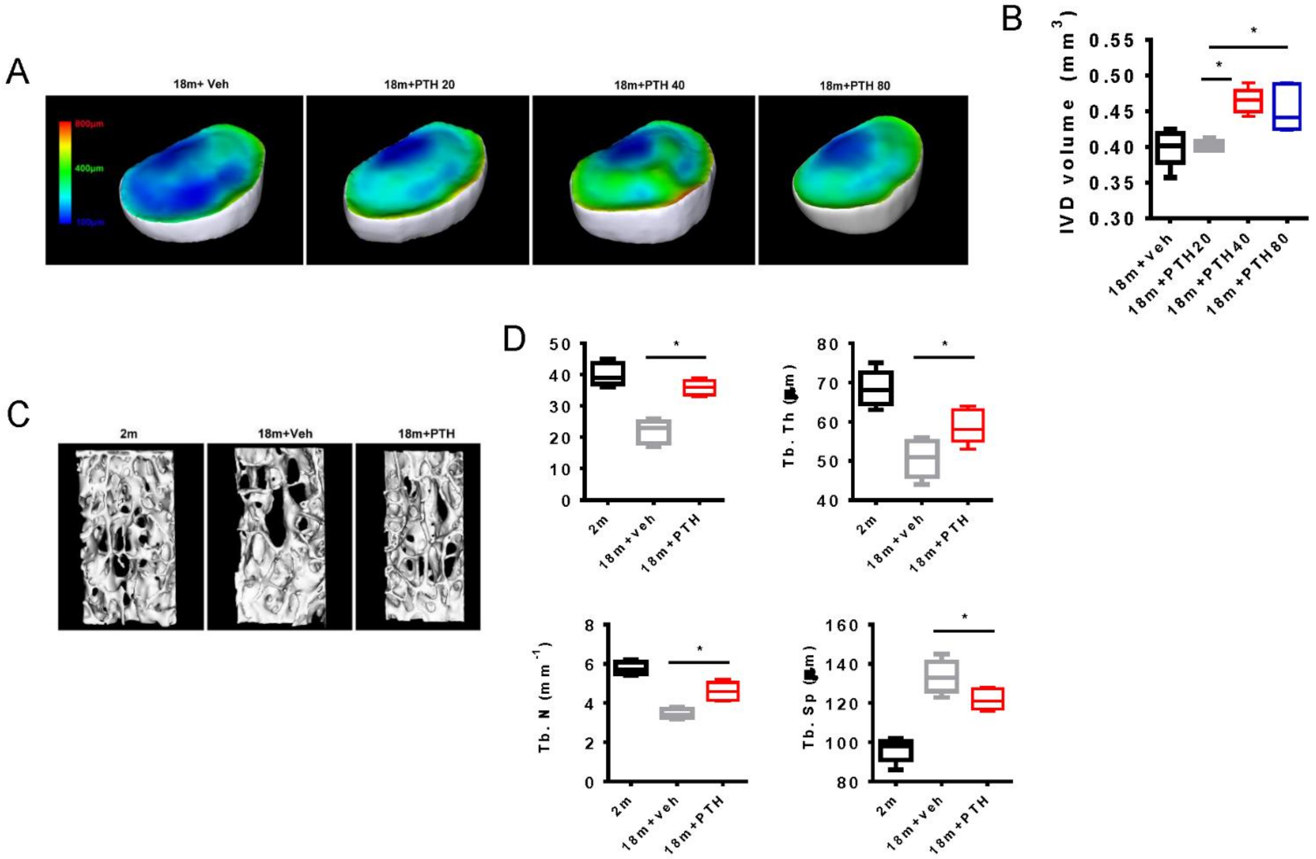

Supplementary Figure 2 The dose screen of intermittent PTH effect on IVD volume. (A) The 3D thickness distribution of IVD with iPTH injection of PTH (1-34) 20 μg/kg/d, 40 μg/kg/d and 80 μg/kg/d, daily for 8 weeks. (B) Quantitative analysis of IVD volume of (A). (C) 3D image of L4 vertebra microarchitecture from 2-month old mice and 18-month old mice with or without iPTH. (D) Quantitative analysis of trabecular bone volume fraction (BV/TV), trabecular thickness (Tb. Th), trabecular number (Tb. N) and trabecular separation (Tb. Sp) with iPTH injection. **P < 0.05*, ***P < 0.01*. Statistical significance was determined by one-way ANOVA and student *t*-test. All data are reported as the mean ± s.d.

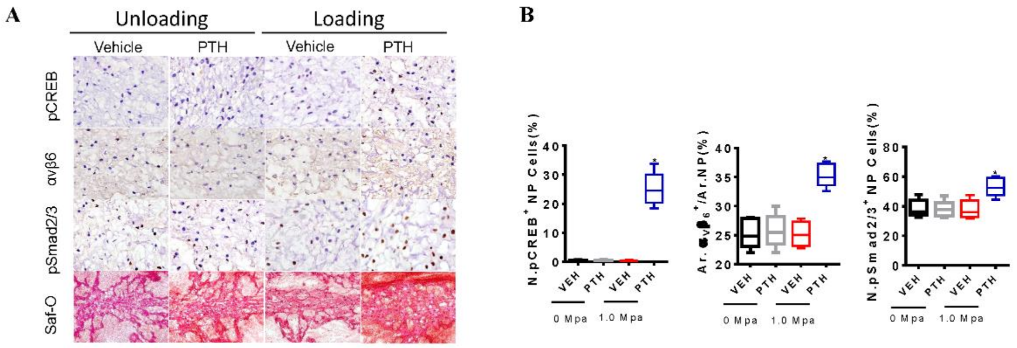

Supplementary Figure 3 The effect of PTH treatment in an IVD ex vivo compression model of 3-month-old rat. (A) pCREB, Integrin $\alpha_V\beta_6$, pSmad2/3 or Safranin-O staining of IVD sections from an IVD ex vivo compression model of 3-month-old rat with treatment of either vehicle or PTH (PTH1-34, 100nM). (B) Quantitative analysis of the percentage of pCREB, pSmad2/3 positive cells and the Integrin αVβ6 positive areas as a percentage of total IVD area (Ar) of (t). All data are reported as the mean ± s.d. *P < 0.05, **P < 0.01. n=8 per group. Statistical significance was determined by one-way ANOVA and student t-test.

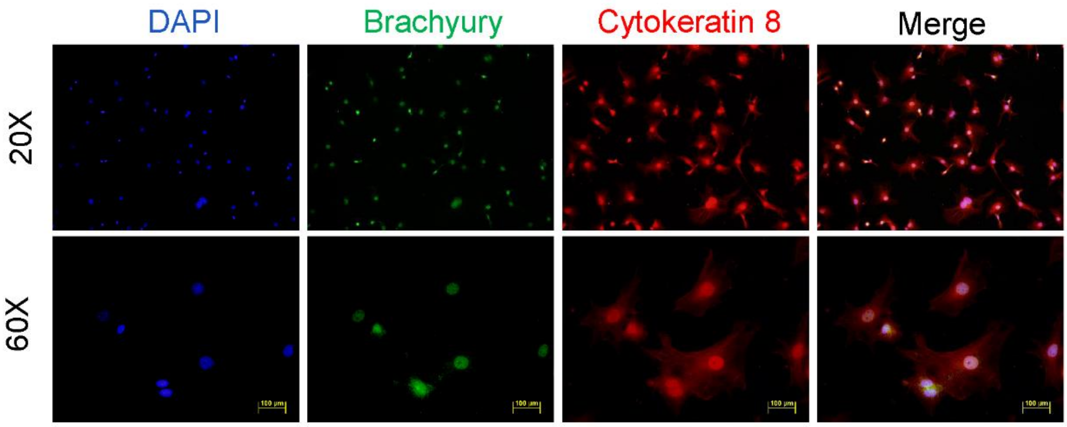

Supplementary Figure 4 Identification of NP Cells from human lumbar disc specimens. Immunostaining for DAPI (blue) and two markers of NP cell (Brachyury (green), Cytokeratin 8 (red)). Scale bar, 100μm.

Supplementary Table 1

Intergrin β6 promoter sequence

Target gene	Forward primer (5'-3')	Reverse primer (5'-3')
Primer 1	TGTGCTGTTCCAACCTCT	TTCCTGAAGAACACCCTG
Primer 2	TTGAAACGAACCCTGAAA	TTCCCTAGCCTTCCTTCT
Primer 3	ATTTTGGTGTAAGTTCTATG	GACTATATTTCTATTGCTGTTGTGA
Primer 4	TCAGCGTTACAAGACCAA	TCCAGGTAGCCTCTGTTT
GAPDH	CCTGCTTATCCAGTCCTAGCTCA	AAATGAGGCGGGTCCAAAG

Supplementary Table 2

The primers sequences

Target gene	Forward primer (5'-3')	Reverse primer (5'-3')
ACAN	CAGATGGCACCCTCCGATAC	GACACACCTCGGAAGCAGAA
CCN2	CCGCCAACCGCAAGATC	ACCGACCCACCGAAGACA
αV	ACTGTGAAGGCGCAGAATCA	TGCCTCTATCCAGTCGACCA
β3	CTCCCTTCTTCCCTCCCCTC	ATCTCGATTACGGGACACGC
β5	GCCAAGTTCCAAAGTGAGCG	CTACCAGGTCCCTTAGGGCT
β6	CTCACGGGTACAGTAACGCA	CCACAAAAGAGCCAAAGCCC
β8	TTATGCAGCAGAGCCCCATC	CAAGACGGAAGTCACGGGAA
GAPDH	AATGTGTCCGTCGTGGATCTGA	AGTGTAGCCCAAGATGCCCTTC
